# High-Dimensional Feature Selection Using Improved Hybrid Breeding Optimization Algorithm with Feature Grouping

**DOI:** 10.3390/biomimetics11060406

**Published:** 2026-06-08

**Authors:** Zhiwei Ye, Yawen Yan, Yujun Ma, Fan Ma, Ting Cai

**Affiliations:** 1 School of Computer Science and Artificial Intelligence, Hubei University of Technology, Wuhan 430068, China; hgcsyzw@hbut.edu.cn (Z.Y.); 102301226@hbut.edu.cn (Y.Y.); 102301198@hbut.edu.cn (F.M.); caiting@hbut.edu.cn (T.C.); 2 Hubei Provincial Key Laboratory of Green Intelligent Computing Power Network, Wuhan 430068, China

**Keywords:** high-dimensional feature selection, feature grouping, hybrid breeding optimization algorithm, symmetric uncertainty, multi-strategy cooperation, minimum redundancy maximum relevance, swarm intelligence, mutual information, simulated annealing, Shannon entropy

## Abstract

Feature selection is essential for improving classification performance in high-dimensional biomedical data, yet conventional metaheuristic algorithms often suffer from premature convergence and loss of population diversity. To address these issues, this paper proposes a Feature Grouping and Improved Hybrid Breeding Optimization framework (FGIHBO). First, the original feature space is hierarchically partitioned using the Maximum Relevance Minimum Redundancy criterion and Symmetric Uncertainty analysis to alleviate the curse of dimensionality. Then, a Multi-Strategy Synergistic Improved Hybrid Breeding Optimization (MSIHBO) algorithm is developed by incorporating Grey Wolf Optimizer (GWO) guidance and a Shannon entropy-adaptive simulated annealing mechanism to balance exploration and exploitation. Experimental results on the CEC2022 benchmark suite demonstrate that MSIHBO provides robust optimization performance across diverse problem categories. Furthermore, evaluations on eleven high-dimensional biomedical datasets show that FGIHBO achieves average classification accuracies ranging from 92.77% to 97.66%. Compared with representative algorithms, including Multi-strategy Improved Grey Wolf Optimizer (MIGWO), Hybrid Whale Optimization Algorithm based on Gathering strategy (HWOAG), Dynamic Crow Search Algorithm (DCSA), GWO, Hybrid Breeding Optimization (HBO), Hybrid Breeding Optimization based on Lévy flight and Elite Opposition-Based Learning strategy (LEHBO), and MSIHBO, the proposed framework improves average classification accuracy by 1.47–27.46%, with the largest gain observed on dataset D10 relative to HWOAG. These results confirm the effectiveness, robustness, and scalability of the proposed framework for high-dimensional biomedical feature selection.

## 1. Introduction

The rapid advancement of information technology has led to an exponential increase in the scale of data and the dimensionality of features in fields such as cybersecurity, biomedicine, and industrial monitoring. Such high-dimensional data typically contain a significant volume of redundant features and noise, which not only elevates the complexity of model construction but also compromises the generalization capability and stability of the resulting systems [1]. More critically, the inherent high sparsity and complex coupling structures of high-dimensional data spaces often cause traditional analysis methods to suffer from the curse of dimensionality [2]. Consequently, the precise extraction of feature subsets that are both representative and highly discriminative from massive raw datasets has become a crucial challenge in overcoming the bottlenecks of high-dimensional data analysis [3].

In the feature selection literature, there is no universally accepted threshold for categorizing low-, medium-, and high-dimensional data. Existing studies generally characterize dimensionality according to both the number of features (*p*) and its relationship to the sample size (*n*). Problems with p≪n are commonly regarded as low-dimensional, whereas cases where *p* is comparable to or significantly larger than *n* (p≫n) are typically considered high-dimensional [4]. The practical interpretation of dimensionality may also vary across application domains. For example, feature spaces containing several thousand dimensions are common in image analysis, while text mining and genomic datasets often involve tens of thousands of features.

Feature Selection (FS), which serves as a critical data preprocessing technique to mitigate the curse of dimensionality [5], aims to isolate a subset of features highly relevant to the target task from the original feature space [6,7]. While preserving the original physical significance and interpretability of the data, this technique effectively eliminates redundant and noisy interference, substantially enhancing the computational efficiency and analytical performance of subsequent models [8,9]. However, high-dimensional FS is inherently a highly complex combinatorial optimization problem [10]. As the number of features increases, the search space of candidate subsets expands exponentially, rendering traditional deterministic search algorithms incapable of identifying the global optimal solution within limited computational resources [1,11].

Metaheuristic optimization algorithms [12,13] have been extensively applied to FS tasks because of their robust global search capabilities and high adaptability to diverse problems [14,15,16,17]. Nevertheless, these traditional algorithms exhibit substantial limitations when navigating the complex search spaces generated by high-dimensional data. First, they typically treat features as mutually independent dimensions and conduct undifferentiated searches, largely disregarding the intrinsic feature correlations and modular group structures inherent in high-dimensional data [18,19]. When massive amounts of redundant and strongly correlated features are tightly coupled, such blind search strategies are easily misled by irrelevant information, leading to severely degraded optimization efficiency [20]. Second, as the dimensionality of the features increases, the search space for candidate subsets expands exponentially. In such excessively high-dimensional spaces, traditional algorithms struggle to maintain effective population diversity. Consequently, their search efficiency drops sharply, and they become highly susceptible to stagnation in local optima, failing to achieve a dynamic balance between extensive global exploration and intensive local exploitation [21,22].

To address the aforementioned limitations of traditional algorithms in high-dimensional FS, this paper proposes a novel method based on Feature Grouping and Improved Hybrid Breeding Optimization (FGIHBO). First, to address the common disregard for feature correlations, a feature grouping strategy is introduced to pre-reconfigure the search space. Through the quantification of inter-feature correlations, the highly coupled original feature space is partitioned into several low-redundancy feature subspaces. This strategy eliminates the blindness inherent in traditional undifferentiated searches and transforms the unordered high-dimensional solution space into a modular structure, effectively reducing the search dimensionality and mitigating the misleading influence of irrelevant information. Second, to enhance the optimization capability in high-dimensional spaces, a Multi-Strategy Synergistic Improved Hybrid Breeding Optimization (MSIHBO) algorithm is developed as the core search optimizer, featuring targeted enhancements to its underlying update mechanisms. By optimizing the dynamic population partitioning and incorporating a multi-strategy subpopulation evolution mechanism, the algorithm’s ability to maintain population diversity is significantly enhanced, thereby achieving a dynamic balance between extensive global exploration and intensive local exploitation. By integrating external spatial dimensionality reduction via grouping with internal search mechanism optimization, this method aims to effectively alleviate the curse of dimensionality while further improving classification accuracy and stability in FS tasks. Specifically, the main contributions of this work are as follows:

- A high-dimensional FS method FGIHBO is proposed. This method deeply integrates the structural decoupling of the external feature space with the mechanism upgrades of the internal optimization algorithm, establishing a comprehensive FS architecture. Experimental results on 11 benchmark datasets demonstrate accuracy improvements of up to 27.46%, highlighting the effectiveness and robustness of the proposed framework for high-dimensional FS.
- A feature grouping and spatial dimensionality reduction strategy based on feature correlation metrics is designed. Information-theoretic indicators are utilized to perform multi-stage filtering and structural grouping of the raw high-dimensional data, effectively eliminating redundant noise and breaking strong feature coupling. This process reconstructs a low-dimensional and well-structured search space for subsequent optimization. Experimental results show that the proposed grouping strategy improves classification accuracy by up to 12.89% over the non-grouped variant, demonstrating its effectiveness in reducing search complexity and enhancing feature subset quality.
- The MSIHBO algorithm is developed. By reconstructing the underlying evolutionary mechanisms of the base algorithm, this paper introduces a dynamic population partitioning strategy. Adaptive update strategies are specifically designed for different subpopulations to enhance global exploration and reinforce local exploitation, significantly improving the algorithm’s optimization performance in complex high-dimensional spaces and effectively preventing entrapment in local optima. Experimental comparisons indicate that MSIHBO achieves up to 2.30% higher classification accuracy than the original Hybrid Breeding Optimization while maintaining faster convergence and stronger resistance to premature convergence.

The remainder of this paper is organized as follows. Section 2 reviews the related work concerning feature grouping and metaheuristic optimization algorithms. Section 3 introduces the preliminaries of the standard Hybrid Breeding Optimization (HBO) algorithm. Section 4 presents the proposed FGIHBO framework and its evolutionary mechanisms. Section 5 describes the experimental settings and provides a comprehensive analysis of the results. Finally, Section 6 concludes the paper.

## 2. Related Work

This section systematically reviews contemporary research related to our work. Specifically, we examine the recent advancements and limitations of two foundational topics: feature grouping methods for high-dimensional data, and metaheuristic optimization algorithms for FS.

### 2.1. Feature Grouping Methods for High-Dimensional Data

In fields such as cybersecurity and biomedicine, the processing of high-dimensional data often involves a feature dimensionality that significantly exceeds the number of available samples, exhibiting the typical characteristics of high dimensionality and small sample sizes [13]. Such data not only contain a substantial volume of irrelevant noise but also possess complex correlations and redundant structures among features. Traditional FS methods generally treat each feature as an independent evaluation unit for a full-space search, thereby severely neglecting the modular group attributes inherent in the data [23,24]. Feature Grouping (FG) [25] techniques aim to partition the original high-dimensional space into several low-coupling feature subspaces by measuring the correlations between features [26]. This divide-and-conquer strategy effectively breaks down the redundant entanglements among features, leading to a significant reduction in both the dimensionality of the solution space and the overall optimization complexity [27].

The core of implementing FG lies in the establishment of reasonable criteria for measuring feature correlations. Commonly employed methodologies primarily include distance-based metrics [28], linear correlation measures (e.g., Pearson correlation coefficient) [29], and non-linear metrics derived from information theory [30]. Given that high-dimensional data often involve complex non-linear interactions between features, traditional linear metrics are frequently insufficient to accurately characterize the true distribution of the data. Consequently, information-theoretic indicators [31], such as Mutual Information (MI) [32,33,34] and Symmetric Uncertainty (SU) [35], have gained more extensive adoption in FG research [36]. Specifically, SU can effectively capture non-linear associations between features while eliminating the computational bias resulting from variations in feature value ranges, proving to be a robust tool for feature correlation evaluation [37].

Based on the established correlation measurement criteria, academic research has proposed diverse FG strategies. Currently, the prevalent methodologies include clustering-based grouping [38] and ranking-based partitioning strategies [39]. Clustering-based approaches form feature clusters by maximizing intra-group correlation and minimizing inter-group correlation [40,41]. In contrast, ranking-based strategies systematically partition the feature space into a predetermined number of modules based on the relevance scores between features and the target variable [42]. The ultimate objective of these strategies is to ensure that features within the same group exhibit high functional similarity or redundancy, while features across different groups remain as independent as possible.

The introduction of FG mechanisms holds significant application value for high-dimensional FS tasks. Once the vast and disordered high-dimensional space is pre-decoupled into well-structured low-dimensional subspaces [26], subsequent optimization algorithms can perform structured searches guided by grouping information. This paradigm not only effectively circumvents the misleading influence of massive redundant features on the search direction but also enables the algorithm to adopt differentiated optimization strategies tailored to the characteristics of different feature groups. Consequently, it establishes a solid foundation for enhancing both the classification performance and search efficiency of high-dimensional FS models.

### 2.2. Feature Selection Based on Metaheuristic Algorithms

FS is inherently a complex combinatorial optimization problem. As the feature dimensionality increases, the search space expands exponentially, rendering traditional exhaustive search or deterministic algorithms computationally prohibitive [18]. Metaheuristic algorithms have emerged as effective tools for solving such NP-hard problems because of their gradient-free nature, global optimization capabilities, and high flexibility [43].

In FS tasks, metaheuristic algorithms typically encode each feature subset as a binary solution vector [44]. To date, a variety of representative algorithms have been successfully deployed in this domain, including the Genetic Algorithm (GA) [45], Particle Swarm Optimization (PSO) [46], Grey Wolf Optimizer (GWO) [47], and the Whale Optimization Algorithm (WOA) [48]. By incorporating diverse encoding schemes and fitness functions, researchers have empowered these algorithms to automatically identify feature combinations with high discriminative power.

However, in high-dimensional data scenarios, such as gene expression profiles and large-scale image features, traditional metaheuristic algorithms face severe challenges. Due to the vast scale and complex distribution of the search space, these algorithms frequently exhibit significant performance degradation during the late stages of optimization. On the one hand, they are highly susceptible to becoming trapped in local optima within the expansive discrete space, making it difficult to escape sub-optimal regions. On the other hand, as the search scope expands, the capacity of these algorithms to balance global exploration and local exploitation diminishes, resulting in slow convergence and degraded search efficiency. Therefore, designing more efficient optimization mechanisms to address the challenges posed by ultra-high-dimensional feature spaces remains an active research hotspot in this field.

To provide a clear and intuitive contrast of the methods reviewed above, a comprehensive comparison summarizing the methodologies, key results, and structural limitations of these existing approaches is presented in Table 1. This comparison directly highlights the open challenges that our proposed framework aims to address.

## 3. Preliminaries

### Standard Hybrid Breeding Optimization Algorithm

HBO algorithm, proposed by Ye et al. in 2016, is a swarm intelligence optimization technique inspired by the biological mechanism of the three-line hybrid rice breeding system in China [49]. By simulating the processes of hybridization, selfing, and renewal updating inherent in rice breeding, the algorithm facilitates information exchange and cooperative evolution among three functional subpopulations: the maintainer, restorer, and sterile lines. This multi-population structure enables the algorithm to effectively enhance global search capabilities while maintaining population diversity, thereby alleviating common issues such as premature convergence and low search efficiency to achieve global optimization within complex solution spaces [50,51,52].

In the standard HBO algorithm, the partitioning of the population relies strictly on the fitness ranking of the individuals. Assuming a total population size of *N*, in each iteration, the algorithm first evaluates the fitness of all individuals and sorts them based on their fitness values from best to worst. Subsequently, the top one-third of the individuals are classified as the maintainer line, the bottom one-third as the sterile line, and the middle one-third as the restorer line. Following the population partitioning, the algorithm updates positions through the following three core steps:

(1) Hybridization: The hybridization operation primarily occurs between the maintainer line and the sterile line. To improve the search quality of inferior individuals, an individual from the sterile line interacts with a randomly selected individual from the maintainer line for information exchange, thereby generating a new candidate solution integrating the positional advantages of both. The position update is formulated as follows:

(1) $$X_{new}=\frac{r_{1}X_{M,j}^{t}+r_{2}X_{S,i}^{t}}{r_{1}+r_{2}},$$

where $X_{S,i}^{t}$ represents the *i*-th sterile line individual at the *t*-th iteration; $X_{M,j}^{t}$ denotes the randomly selected *j*-th maintainer line individual; and $r_{1}$ and $r_{2}$ are random numbers uniformly distributed within the range of [−1,1]. After generating the new individual $X_{new}$, the algorithm evaluates its fitness. If the fitness of the new individual is superior to that of the original sterile line individual $X_{S,i}^{t}$, the original individual is replaced by the new one in the next iteration (i.e., $X_{S,i}^{t+1}=X_{new}$); otherwise, the original individual is retained.

(2) Selfing: The selfing operation functions exclusively within the restorer line, primarily aiming to conduct local exploitation around current high-quality solutions. To maintain population diversity while converging towards the global optimum, this operation introduces perturbations from random individuals during the position update. By applying the distance vector between the global best individual and a randomly selected individual within the restorer line to the current individual, a new candidate individual is generated. The mathematical expression is formulated as follows:

(2) $$X_{new}=X_{R,i}^{t}+r_{3}\left(X_{best}^{t}-X_{R,j}^{t}\right),$$

where $X_{R,i}^{t}$ represents the current restorer line individual being updated; $X_{R,j}^{t}$ denotes another randomly selected individual from the restorer line (satisfying j≠i); $X_{best}^{t}$ is the global best individual in the current population; and $r_{3}$ is a uniformly distributed random number in the range of [0,1]. Similarly, if the fitness of $X_{new}$ is superior to that of the original individual $X_{R,i}^{t}$, the original one is replaced in the next iteration (i.e., $X_{R,i}^{t+1}=X_{new}$); otherwise, the original individual is retained, and a selfing failure counter is incremented by 1. This mechanism establishes the triggering condition for the subsequent renewal operation.

(3) Renewal: If certain restorer line individuals fail to discover a superior fitness value after multiple consecutive selfing attempts (i.e., the selfing failure counter reaches the predefined upper limit max_self), it indicates that these individuals have likely stagnated in local optima. In such cases, the algorithm triggers the renewal mechanism to preserve population diversity. The renewal equation is formulated as follows:

(3) $$X_{R,i}^{t+1}=X_{R,i}^{t}+lb+r_{4}\left(ub-lb\right),$$

where $X_{R,i}^{t+1}$ and $X_{R,i}^{t}$ denote the position vectors of the *i*-th restorer line individual at the (t+1)-th and *t*-th iterations, respectively; ub and lb represent the upper and lower bounds of the search space; and $r_{4}$ is a uniformly distributed random number in the range of [0,1]. By introducing large-scale random perturbations based on the current position, the renewal operation compels the individual to escape from local optima. Upon completion of the renewal, the selfing counter for this individual is reset to zero, and the selfing optimization process is reinitiated.

Despite the inherent advantages of multi-population cooperative evolution in the standard HBO algorithm, it still exhibits pronounced limitations when addressing high-dimensional FS problems. On the one hand, its three-line population partitioning mechanism, which relies entirely on static rankings, is overly inflexible, frequently leading to the premature elimination of high-quality individuals located near the search boundaries. On the other hand, its relatively simplistic hybridization and selfing update mechanisms struggle to achieve an effective balance between global exploration and local exploitation within the exponentially expanding high-dimensional search space. Consequently, there is an urgent need for deep reconstruction and enhancement of the underlying operational mechanisms of the standard HBO algorithm.

The theoretical concepts introduced in this section establish the foundation for the proposed framework detailed in Section 4. While the classical three-line breeding structure serves as the architectural backbone of our MSIHBO optimizer (Section 4.3), its conventional mechanisms are substantially reconstructed to tackle the unique challenges of high-dimensional FS. Specifically, the maintainer line, which traditionally acts solely as a static genetic donor in the standard algorithm, is advanced into an active GWO-guided encircling mechanism (Equation (14), Section 4.3.2). Furthermore, the traditional sterile line operation (Equation (1)) is evolved into a Shannon entropy-adaptive simulated annealing strategy (Section 4.3.3). Conversely, the restorer line strictly retains the standard selfing (Equation (2)) and renewal (Equation (3)) mechanisms to preserve the fundamental local exploitation properties of the biological heuristic. These operators, along with the foundational fitness evaluation concept, are ultimately integrated into a specific wrapper-based objective function (Equation (23), Section 4.4.2).

## 4. The Proposed Method

### 4.1. Overall Execution Workflow of the Proposed FGIHBO

High-dimensional data are replete with massive redundant information and irrelevant features. If metaheuristic algorithms are directly employed to conduct blind searches across the entire feature space, they are highly prone to triggering the curse of dimensionality, which leads to degraded optimization efficiency and severe vulnerability to local optima stagnation. To break through this bottleneck, this paper proposes the FGIHBO method. Adhering to the design philosophy of dimensionality reduction and decoupling first, followed by cooperative optimization, the core execution workflow is structured into two sequential phases.

**Figure 1:**
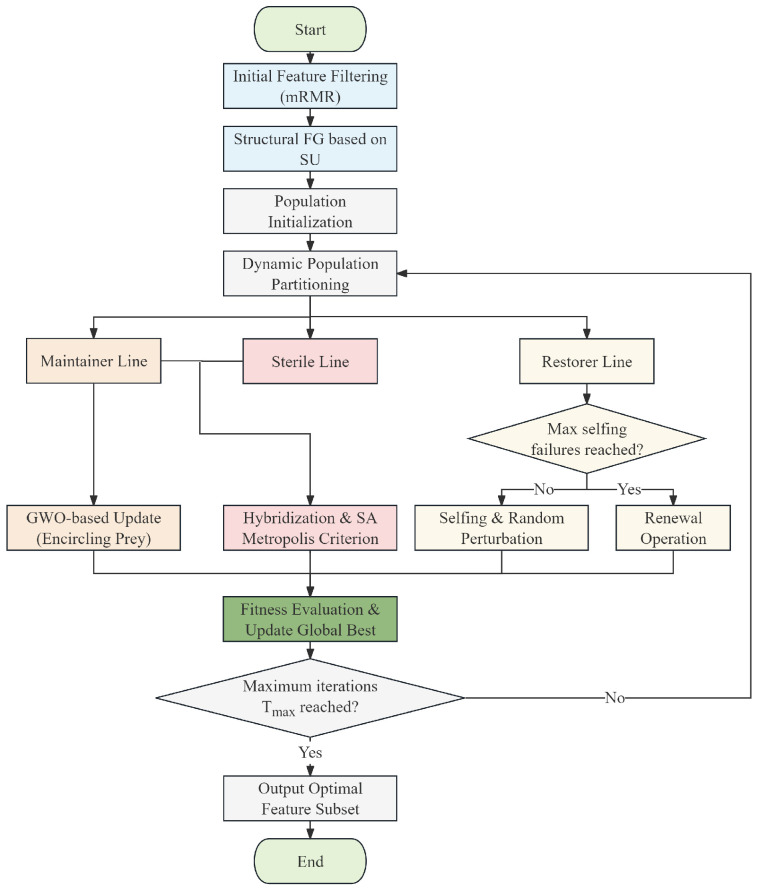
The overall execution workflow of the proposed FGIHBO.

The first phase achieves structural dimensionality reduction of the high-dimensional space through feature filtering and grouping. Subsequently, the second phase leverages a multi-population cooperative evolutionary mechanism to conduct precise optimization within the reconstructed subspaces. The specific execution steps of the algorithm are detailed as follows, and the complete execution workflow is illustrated in Figure 1:

Step 1: Initial feature filtering. For the raw ultra-high-dimensional data, the MI between each feature and the target label is first calculated. The maximum relevance minimum redundancy (mRMR) criterion is then utilized for preliminary screening to eliminate the vast majority of irrelevant noise features, thereby achieving an initial reduction of the search space.

Step 2: Structural FG based on SU. For the feature set retained after the initial screening, SU is employed to measure the degree of non-linear association between features. Based on correlation ranking, highly coupled features are partitioned into the same group. This strategy reconstructs the originally disordered high-dimensional solution space into *M* low-redundancy feature subspaces.

Step 3: Population initialization and dynamic partitioning. The core parameters of the improved hybrid breeding algorithm are initialized. An initial population is generated within the low-dimensional subspaces partitioned in Step 2, and the fitness value of each individual is evaluated. Subsequently, a roulette wheel selection mechanism is implemented to break the traditional fixed-ratio constraints, dynamically partitioning the population into three distinct subpopulations: the maintainer line, the restorer line, and the sterile line.

Step 4: Multi-strategy synergistic evolutionary optimization. In each iteration, the three subpopulations execute customized evolutionary strategies:

1. Maintainer line update: The encircling prey mechanism from GWO is introduced to facilitate information exchange among elite individuals. This proactive evolution strategy prevents the top-performing individuals from stagnating, significantly enhancing the algorithm’s local exploitation precision and convergence speed.
2. Restorer line update: The selfing operation, augmented with random distance perturbations, is executed to conduct continuous local search. If an individual’s consecutive selfing failures reach a predefined upper limit, the renewal operation is triggered to forcefully escape local optima traps.
3. Sterile line update: Individuals in the sterile line hybridize with those in the maintainer line. The Metropolis acceptance criterion from Simulated Annealing (SA) is incorporated during this process. By probabilistically retaining newly generated inferior individuals, this mechanism significantly enhances population diversity and improves overall search efficiency in high-dimensional spaces.

Step 5: Fitness evaluation and termination condition verification. Fitness evaluation is performed on all updated individuals, and the current global best solution is recorded and updated. If the current iteration number reaches $T_{max}$, the algorithm terminates and outputs the feature subset with the highest fitness as the final FS result; otherwise, the process returns to Step 3 to continue the iteration.

### 4.2. Information-Theoretic Feature Space Dimensionality Reduction and Grouping Strategy

When confronting high-dimensional or ultra-high-dimensional datasets, features frequently exhibit complex non-linear interactions coupled with a substantial amount of redundant noise. To elevate the search efficiency and convergence speed of the FGIHBO algorithm, this paper designs an information-theoretic feature space reconstruction strategy prior to the execution of population evolution. Initially, the mRMR criterion is applied to perform a coarse-grained compression of the feature space. Subsequently, SU and a bucketing mechanism are utilized to achieve the fine-grained structural grouping of features.

#### 4.2.1. Initial Feature Filtering Based on mRMR

To reduce the dimensionality of the solution space for the subsequent heuristic search, this paper introduces the mRMR feature filtering strategy in the initial phase. Let the feature space of the original high-dimensional dataset be denoted as $F=\{f_{1},f_{2},\ldots ,f_{D}\}$ (where *D* represents the original feature dimensionality), and the target class label be *C*. The core philosophy of mRMR is to maximize the relevance between candidate features and the class label while simultaneously minimizing the redundancy among the selected features. During the iterative evaluation process of FS, let the currently selected feature subset be *S*. For any unselected candidate feature $f_{i}\in F-S$, its potential to be selected is comprehensively determined by the following two information-theoretic indicators:

1. Relevance: The MI $I(f_{i};C)$ is utilized to measure the degree of relevance between the candidate feature $f_{i}$ and the class label *C* (target for maximization). MI is defined as follows:
  (4) $$I\left(f_{i};C\right)=\sum_{f_{i}}\sum_{C}p\left(f_{i},C\right)\log\frac{p(f_{i},C)}{p\left(f_{i}\right)p\left(C\right)},$$
  where $p(f_{i})$ and p(C) denote the marginal probability distributions of the feature $f_{i}$ and the class label *C*, respectively; and $p(f_{i},C)$ represents their joint probability distribution.
2. Redundancy Penalty: The term $\frac{1}{\left|S\right|}\sum_{f_{j}\in S}I\left(f_{i};f_{j}\right)$ is employed to measure the information overlap between the candidate feature $f_{i}$ and all previously selected features $f_{j}$ within the set *S* (target for minimization).

Integrating the two aforementioned criteria, the comprehensive mRMR evaluation value for the candidate feature $f_{i}$ is calculated as follows:

(5) $$mRMR\left(f_{i}\right)=I\left(f_{i};C\right)-\frac{1}{\left|S\right|}\sum_{f_{j}\in S}I\left(f_{i};f_{j}\right).$$

Forward Greedy Search: Initially, the selected subset is empty (S=∅). The algorithm begins by selecting the single feature with the maximum relevance to the class label and adding it to *S*. Subsequently, in each iteration, the mRMR score of every remaining candidate feature is calculated, and the feature with the highest score is appended to the set *S*. This iterative process continues until the size of set *S* reaches the predefined retention dimensionality $D^{\prime }$, at which point *S* constitutes the pre-selected feature set $F^{\prime }$ ($F^{\prime }\subset F$, where $D^{\prime }\ll D$).

#### 4.2.2. Structural Feature Grouping Based on Symmetric Uncertainty

Although the feature set $F^{\prime }$ filtered by mRMR is dimensionally reduced, it still retains a certain scale. Directly feeding it into the hybrid breeding algorithm for global optimization is likely to result in inefficient population searches. To address this, this paper employs SU to characterize the non-linear correlations within the feature set $F^{\prime }$, and proposes a structural grouping strategy based on bucket sampling. Concurrently, a Softmax mechanism is utilized to adaptively allocate the initial sizes of the subpopulations.

First, for any feature $f_{i}$ in the filtered feature set $F^{\prime }$ and the class label *C*, their MI $I(f_{i};C)$ alongside their respective information entropies $H(f_{i})$ and H(C) are calculated. The information entropy is defined as follows:

(6) $$H\left(f_{i}\right)=-\sum_{f_{i}}p\left(f_{i}\right)\log p\left(f_{i}\right),\;H\left(C\right)=-\sum_{C}p\left(C\right)\log p\left(C\right).$$

While the preceding mRMR stage utilizes unnormalized MI for sequential greedy filtering, its dynamic scores are heavily dependent on the previously selected subset and cannot serve as an absolute global ranking metric. To address this, the SU indicator is introduced to eliminate the scale bias caused by the varying value ranges of different features, normalizing the correlation metric to the interval [0,1]. By establishing this standardized, independent baseline, the SU evaluation provides the additional structural information, specifically a quantifiable correlation gradient, which is strictly required for the subsequent unbiased bucket partitioning, as formulated below:

(7) $$SU\left(f_{i},C\right)=2\times \frac{I(f_{i};C)}{H\left(f_{i}\right)+H\left(C\right)}.$$

After calculating the SU values for all retained features, the structural FG and resource allocation are executed. The specific logic is detailed as follows:

Bucket-based Sorting and Partitioning: Suppose the feature set $F^{\prime }$ needs to be divided into *M* feature subspaces. First, the number of features allocated to each subspace is calculated as $num=\lfloor D^{\prime }/M\rfloor$. All features are then sorted in descending order according to their SU values and sequentially evenly divided into num “buckets”, where each bucket contains exactly *M* features (i.e., $F^{\prime }=B_{1}\cup B_{2}\cup \ldots \cup B_{num}$). Although $SU(f_{i},C)$ explicitly evaluates feature-label relevance, this sorting process indirectly facilitates inter-feature decoupling. Since mutually correlated features typically exhibit similar SU values, they tend to fall into the same bucket and are subsequently distributed into different subspaces, thereby effectively mitigating intra-subspace redundancy.

Intra-group Heterogeneous Sampling: For the *m*-th feature subspace $G_{m}$ (m∈[1,M]), the algorithm randomly extracts one feature from each bucket to append to this subspace until its construction is complete. Unlike conventional random stratified partitioning that divides features blindly, this mechanism performs a structural feature grouping. Since the buckets are strictly quantified and ordered by SU, sampling across these ordered buckets ensures that each subspace $G_{m}$ systematically inherits the correlation gradient (i.e., a heterogeneous mixture of strongly, moderately, and weakly correlated features) of the global space. This structurally aware extraction preserves the underlying information distribution and effectively circumvents the redundancy caused by the clustering of highly correlated features, thereby substantially enhancing the genetic diversity within each subspace.

Computational Resource Allocation Based on Softmax: To enable the subsequent improved hybrid breeding algorithm to allocate computational power more rationally, the Feature Importance (FI) of the *m*-th subspace is defined as the cumulative sum of the SU values of all its internal features:

(8) $$FI\left(G_{m}\right)=\sum_{k=1}^{num}SU\left(f_{m,k}^{\prime }\right).$$

To widen the resource allocation gradient between superior and inferior subspaces, this paper employs the Softmax exponential function. Unlike linear proportional allocation strategies, the exponential nature of Softmax heavily amplifies marginal differences in subspace importance $FI(G_{m})$. This theoretically ensures that highly correlated subspaces receive substantially more computational resources, while mathematically preventing any inferior subspace from being allocated zero individuals, thereby robustly maintaining global structural diversity. The allocated population size $N_{m}$ for the subspace $G_{m}$ is calculated as follows:

(9) $$N_{m}=\mathrm{round}\left(N\times \frac{e^{FI(G_{m})}}{\sum_{j=1}^{M}e^{FI(G_{j})}}\right),$$

where round(·) denotes the rounding function. Through the exponential amplification effect, this mechanism transforms marginal differences in subspace importance into significant variations in the number of population individuals. This effectively guides the algorithm to focus more computational resources on superior subspaces containing crucial features.

### 4.3. Multi-Strategy Synergistic Improved Hybrid Breeding Optimization Algorithm

When tackling high-dimensional FS problems, the standard HBO algorithm exhibits several inherent limitations, including an uneven initial population distribution, an inflexible population partitioning mechanism, a lack of evolutionary capacity within the maintainer line, and the high susceptibility of the sterile line to becoming trapped in local optima. To comprehensively elevate the search efficiency and convergence accuracy within the partitioned subspaces, this paper conducts a systematic reconstruction of every evolutionary phase of the standard HBO, proposing an MSIHBO algorithm.

#### 4.3.1. Population Initialization and Dynamic Partitioning Strategy

A high-quality initial population is a foundation for ensuring global search capabilities. To address the issue of uneven sampling in high-dimensional spaces caused by random initialization, this paper first introduces the Latin Hypercube Sampling (LHS) strategy to generate an initial continuous population of size *N*, ensuring spatial space-filling uniformity across all dimensions. Subsequently, an Elite Opposition-Based Learning (EOBL) mechanism is introduced to enhance the population. The elite individual $X_{elite}$, ranked at the top in terms of current fitness, is selected to generate its opposite individual $X_{opp}$ within the search space [lb,ub] utilizing Equation (10):

(10) $$X_{opp}=lb+ub-X_{elite}.$$

The generated opposite individuals are merged with the original population for collective evaluation, and the top *N* individuals with the highest fitness are retained as the final initial population. This significantly broadens the coverage of the initial search range, improving population diversity and the quality of the starting search points from the outset.

Regarding the partitioning of the population structure, the standard HBO employs a fixed three-line method, which frequently leads to the premature elimination of potentially superior individuals located near the boundaries. To overcome this, this paper introduces a probability allocation mechanism based on roulette wheel selection. After sorting in each iteration, the top 1/3 of individuals with the highest fitness are strictly retained as the core maintainer line. For the remaining 2/3 of the individuals, their fitness is first normalized using Equation (11):

(11) $$p_{i}=\frac{f_{i}-f_{min}}{f_{max}-f_{min}+\varepsilon },$$

where $f_{i}$ represents the fitness of the current individual; $f_{max}$ and $f_{min}$ denote the best and worst fitness values among the remaining individuals, respectively; and ε is a minimally small constant to prevent division by zero. Subsequently, a uniformly distributed random number r∈[0,1] is generated. If $r\leq p_{i}$, the individual is dynamically allocated to the restorer line to perform local selfing; otherwise, it is assigned to the sterile line to undergo hybridization. This strategy weakens the rigid partitioning boundaries, enables a smooth transition in the population structure, and effectively maintains diversity during the early stages of population evolution.

#### 4.3.2. Maintainer Line Update Mechanism Integrated with Grey Wolf Optimizer

In the traditional HBO, maintainer line individuals serve solely as providers of superior genetic material and do not undergo position updates themselves, which severely constrains the local exploitation potential of the elite population. To address this, this paper introduces the multi-wolf guiding mechanism from the GWO to conduct targeted evolutionary enhancement on the maintainer line.

In each iteration, the three individuals with the best fitness within the maintainer line are selected and labeled as α, β, and δ, respectively. For any other individual $X_{\omega }$ in the maintainer line, the positional information of these three leader wolves is utilized to cooperatively guide its update. The mathematical expressions are formulated as Equations (12)–(14):

(12) $$D_{k}=\left|C_{k}\cdot X_{k}-X_{\omega }\right|,\;\left(k\in \left\{\alpha ,\beta ,\delta \right\}\right),$$

(13) $$X_{k}^{\prime }=X_{k}-A_{k}\cdot D_{k},\;\left(k\in \left\{\alpha ,\beta ,\delta \right\}\right),$$

(14) $$X_{\omega }^{t+1}=\frac{X_{\alpha }^{\prime }+X_{\beta }^{\prime }+X_{\delta }^{\prime }}{3},$$

where $D_{k}$ represents the distance vector between the current individual $X_{\omega }$ and the leader wolf $X_{k}$; $C_{k}$ is the cooperative swing factor ($C_{k}=2r_{2}$, with $r_{2}$ being a random number in [0,1]), utilized to simulate random perturbations during the search process to evade local optima; $X_{k}^{\prime }$ denotes the candidate position component generated under the guidance of a single leader wolf $X_{k}$; and $A_{k}$ is the convergence factor controlling the balance between global exploration and local exploitation, calculated as $A_{k}=2a\cdot r_{1}-a$ ($r_{1}$ being a random number in [0,1]).

In the standard GWO, the control parameter *a* typically decreases linearly from 2 to 0 with the iteration count. However, this easily leads to premature convergence in the early stages of evolution and insufficient precision in local exploitation during the later stages. To overcome this limitation, this paper introduces a non-linear decay strategy based on a cosine function, reconstructing the parameter *a*:

(15) $$a=\left(a_{max}-a_{min}\right)\cdot \cos\left(\frac{\pi }{2}{\left(\frac{t}{T_{max}}\right)}^{2.5}\right),$$

where $a_{max}$ and $a_{min}$ are the initial and final continuous boundaries of the exploration control parameter, respectively. To match the operational range of the integrated multi-wolf guidance mechanism and ensure an extensive exploration area in the early generations, $a_{max}$ is set to 2, while $a_{min}$ is set to 0 to enforce intensive local refinement at the end of the search. The variable *t* indicates the current iteration index, and $T_{max}$ represents the predefined maximum number of iterations. This non-linear decay strategy enables *a* to maintain a relatively high value during the early stages of iteration to enhance global exploration, while dropping precipitously in the later stages to lock precisely onto the optimal solution region. The exponent of 2.5 is specifically mathematically calibrated to flatten the initial decay curve, thereby strictly extending the global exploration phase, while steepening the subsequent decline to accelerate terminal convergence.

#### 4.3.3. Sterile Line Update Mechanism Integrated with Simulated Annealing and Shannon Entropy

Given their inferior fitness, individuals in the sterile line bear the crucial responsibility of escaping local optima and expanding the search space. In the traditional hybridization operation, if the newly generated individual possesses inferior fitness compared to the original one, it is directly discarded, causing the algorithm to lose valuable trial-and-error opportunities. To address this, after generating the candidate solution $X_{new}$ through hybridization, this paper introduces the Metropolis acceptance criterion from SA alongside a dynamic temperature control mechanism based on Shannon Entropy.

If the candidate solution $X_{new}$ is inferior to the original individual $X_{S,i}^{t}$, the probability $P_{accept}$ determines whether to accept this inferior solution:

(16) $$P_{accept}=\exp\left(\frac{f\left(X_{new}\right)-f\left(X_{S,i}^{t}\right)}{T^{t}}\right),$$

where, f(·) denotes the fitness evaluation function; $X_{S,i}^{t}$ represents the original *i*-th individual in the sterile line at the current iteration; $X_{new}$ is the newly generated candidate solution; and $T^{t}$ is the current simulated annealing temperature. It should be noted that standard SA is conventionally designed for minimization problems, employing the Metropolis criterion P=exp(−ΔE/T), where the energy variation ΔE>0 for an inferior solution. However, since our FS task is fundamentally formulated as a maximization problem, generating an inferior candidate inherently yields a negative fitness increment, i.e., $\Delta f=f\left(X_{new}\right)-f\left(X_{S,i}^{t}\right)<0$. By directly formulating the acceptance probability as $P_{accept}=\exp\left(\Delta f/T^{t}\right)$ without an explicit minus sign, this mechanism mathematically guarantees $P_{accept}\in \left(0,1\right)$ while strictly preserving the core thermodynamic logic of SA: (1) Quality-aware penalty: When the fitness degradation is minor (Δf is a small negative value), the penalty is slight, yielding a higher acceptance probability; conversely, severe degradation leads to an exponentially decaying probability. (2) Temperature-driven exploitation: At high temperatures (large $T^{t}$), the probability remains high to strongly promote global diversity, whereas as $T^{t}$ cools, the probability rapidly converges to 0, enforcing strict local exploitation. Thus, this formulation robustly adapts the Metropolis criterion to the maximization landscape.

To endow the annealing process with adaptive adjustment capabilities, this paper utilizes Shannon Entropy to capture the real-time evolutionary state of the diversity distribution of the sterile line population within the search space. In FS problems, population individuals are represented by binary encoding (a value of 1 indicates the feature is selected, while 0 indicates it is not). By calculating the probabilities of the current sterile line population taking values of 1 and 0 in the *j*-th dimension (denoted as $p_{j}^{1}$ and $p_{j}^{0}$, respectively), the information entropy $H_{j}$ for this dimension is calculated as expressed in Equation (17):

(17) $$H_{j}=-\left(p_{j}^{0}\log_{2}p_{j}^{0}+p_{j}^{1}\log_{2}p_{j}^{1}\right).$$

When $p_{j}^{1}$ and $p_{j}^{0}$ approach 0.5, $H_{j}$ reaches its maximum value of 1, indicating that the population exhibits the greatest divergence in the selection of this feature, and the structure demonstrates high diversity. Conversely, when the probabilities approach 0 or 1, $H_{j}$ approaches 0, indicating that the population has reached a high degree of consensus (i.e., convergence has occurred) in this dimension. Subsequently, the average information entropy $H_{avg}$ across all $D^{\prime }$ dimensions is calculated as an indicator of the overall population dispersion:

(18) $$H_{avg}=\frac{1}{D^{\prime }}\sum_{j=1}^{D^{\prime }}H_{j}.$$

To achieve a dynamic balance where high diversity delays cooling to maintain global exploration, while low diversity accelerates cooling to expedite local convergence, this paper designs an adaptive cooling factor λ based on the $H_{avg}$, which is utilized to update the global temperature parameter $T^{t}$:

(19) $$\lambda =\frac{1}{1+H_{avg}},$$

(20) $$T^{t+1}=\max\left(T^{t}\cdot \left(1-\lambda \right),T_{min}\right).$$

Through this mechanism, population diversity directly regulates the cooling rate of the simulated annealing process. When the sterile-line population remains highly diverse ($H_{avg}$ approaching 1), the adaptive factor λ reaches its lower bound, leading to a slower temperature decay and maintaining a relatively high probability of accepting inferior solutions. This behavior encourages continuous exploration and helps preserve population diversity. As the population gradually converges ($H_{avg}$ approaching 0), λ increases, resulting in a faster temperature reduction and a rapid decrease in the acceptance probability of inferior solutions. Consequently, the search process naturally shifts toward intensive local exploitation. By coupling Shannon entropy with temperature adaptation, the proposed mechanism dynamically balances exploration and exploitation according to the evolutionary state of the population. In summary, to comprehensively address the challenges of high-dimensional FS, the proposed framework integrates traditional foundations with novel mechanism reconstructions. While the underlying three-line (maintainer, restorer, and sterile lines) and standard selfing operators follow the classical HBO philosophy [53], the remaining operational components are entirely reconstructed. Specifically, the external space partition relies on a newly designed information-theoretic bucket sampling mechanism, whereas the internal optimization mechanism incorporates innovative dynamic roulette partitioning, GWO leader guidance, and Shannon entropy-guided temperature adaptation strategy. Table 2 provides a clear component-wise comparison to explicitly distinguish our proposed methodologies from the standard baseline.

### 4.4. Discrete Evaluation Strategy and Computational Complexity

#### 4.4.1. Discretization Strategy Based on Sigmoid Probability Mapping

Native metaheuristic algorithms (including GWO and HBO) typically perform heuristic optimization within continuous real-valued spaces. However, FS is fundamentally an NP-hard discrete combinatorial optimization problem, where each dimension within the solution space possesses only two states: selected (1) and unselected (0).

To resolve this spatial mismatch issue, the proposed algorithm architecturally maintains a dual-coordinate tracking mechanism for each individual: a continuous position vector and a corresponding discrete feature mask. The algorithmic search behaviors (e.g., leader wolf guidance, SA perturbations) are entirely executed within the continuous coordinate system. However, prior to conducting any fitness evaluation, a Sigmoid transfer function coupled with a probability threshold mechanism is employed to dynamically map the updated continuous positions into binary discrete vectors.

Specifically, let the position of the *i*-th individual in the *d*-th dimension of the continuous space during the *t*-th iteration be denoted as $x_{i}^{d}\left(t\right)$. First, the Sigmoid function is utilized to map it into the probability interval of [0,1]. The calculation is formulated as Equation (21):

(21) $$S\left(x_{i}^{d}\left(t\right)\right)=\frac{1}{1+e^{-x_{i}^{d}\left(t\right)}},$$

where $S(x_{i}^{d}\left(t\right))$ represents the probability propensity of the feature in this dimension being selected. Subsequently, a uniformly distributed random number generation mechanism is introduced to discretize the continuous probability into a binary bit $X_{i}^{d}\left(t\right)$ consisting of 0 or 1. The decision rule is as follows:

(22) $$X_{i}^{d}\left(t\right)=\left\{\begin{array}{cc} 1, & \mathrm{if}\;rand<S(x_{i}^{d}\left(t\right))\\ 0, & \mathrm{otherwise} \end{array}\right.,$$

where rand∈[0,1] is a pseudo-random number independently generated during each mapping. When $X_{i}^{d}\left(t\right)=1$, it indicates that the feature corresponding to this dimension is retained to construct the classification model; otherwise, it is eliminated. This probabilistic mapping mechanism permits minute perturbations in the continuous space to be smoothly reflected in the variations of feature combinations, thereby effectively circumventing search stagnation caused by rigid threshold truncation. It is important to emphasize that throughout the evolutionary operations (e.g., selection, hybridization, and GWO updates), the continuous position vector is strictly retained as the persistent genetic material passed to the next iteration. The binary feature mask serves solely as a temporary phenotype for fitness evaluation and is discarded immediately post-evaluation. This design ensures the mathematical continuity and validity of the spatial update equations in subsequent generations.

#### 4.4.2. Fitness Evaluation Mechanism

The FS task inherently encompasses two competing optimization objectives: maximizing classification accuracy and minimizing the number of retained features. Adopting a wrapper-based paradigm, this paper utilizes the K-Nearest Neighbors (KNN) classifier under cross-validation as the core evaluation criterion. KNN is selected as the sole baseline evaluator because of its non-parametric nature and absence of complex internal weight training. This effectively prevents the conflation of the classifier’s own learning capabilities with the inherent discriminative power of the selected features, ensuring that the fitness score strictly reflects the objective quality of the subset. The fitness function is formulated as Equation (23):

(23) $$Fitness=\gamma \times Acc_{KNN}+\left(1-\gamma \right)\times \left(1-\frac{\left|S\right|}{D}\right),$$

where $Acc_{KNN}$ represents the classification accuracy of the selected feature subset evaluated by the KNN classifier, and |S| denotes the cardinality of the selected feature subset (i.e., the total count of active bits in the binary feature mask $X_{i}$). The parameter *D* is a constant denoting the total feature dimensionality of the original dataset, and γ∈[0,1] acts as a vital balancing factor regulating the trade-off between classification accuracy and feature reduction. Given that biomedical pattern recognition demands a strict intolerance for accuracy degradation, this paper prioritizes classification quality by setting γ=0.99, ensuring that feature minimization functions as a secondary regularization penalty without compromising model performance.

#### 4.4.3. Algorithm Time and Space Complexity Analysis

The time complexity of the proposed FGIHBO algorithm primarily consists of three components: population initialization, multi-strategy position update, and fitness evaluation. In traditional global optimization algorithms, the complexity of a single position update for the population is $O(N\times D^{\prime })$, where *N* represents the total population size and $D^{\prime }$ is the pre-selected feature dimensionality after filtering. In contrast, by introducing the feature decoupling and grouping strategy, the search space $D^{\prime }$ is partitioned into *M* subspaces, which significantly reduces the computational overhead of a single cooperative evolutionary position update across all subspaces to $O(N\times \frac{D^{\prime }}{M})$. Assuming the overhead of evaluating a single subset using the KNN classifier is $O(C_{KNN})$ and the maximum number of iterations is $T_{max}$, the overall time complexity of the algorithm is derived as $O(T_{max}\times N\times C_{KNN}+T_{max}\times \frac{N\times D^{\prime }}{M})$. This mathematically demonstrates that the introduction of multi-strategy synergy does not alter the polynomial time characteristic of the algorithm; instead, it substantially alleviates the computational burden brought by the curse of dimensionality through FG.

In terms of space complexity, the FGIHBO algorithm primarily maintains two core data structures: the continuous position matrix for evolutionary optimization and the discrete binary mask matrix for fitness calculation. These structures collectively require a memory footprint of $O(N\times D^{\prime })$. Additionally, recording auxiliary variables such as the positions of the leader wolves (α,β,δ), the historical best solution, and the adaptive temperature parameter $T^{t}$ only necessitates a marginal auxiliary space of the $O(D^{\prime })$ level. Consequently, the overall space complexity is maintained at the $O(N\times D^{\prime })$ level. This linear memory utilization characteristic ensures the model’s exceptional robustness even when processing high-dimensional datasets. Guided by this theoretical robustness, the subsequent empirical validations shift the focus toward computational efficiency. Specifically, the $O(N\times D^{\prime })$ space complexity signifies that even in ultra-high-dimensional scenarios, the algorithm’s memory footprint is merely a few megabytes, which fundamentally poses no physical bottleneck. Furthermore, the subsequent Section 5 systematically presents actual runtime comparisons and scalability evaluations across datasets with exponentially increasing feature dimensions. Compared to the baselines, the proposed grouping mechanism substantially reduces the empirical computational overhead, perfectly corroborating the theoretical time complexity reduction from $O(N\times D^{\prime })$ to $O(N\times \frac{D^{\prime }}{M})$ derived above.

## 5. Experimental Results and Discussion

To verify the effectiveness and robustness of the proposed method when processing high-dimensional biomedical data, this section selects several representative ultra-high-dimensional datasets to conduct FS simulation experiments. The experiments aim to systematically evaluate the performance of the proposed MSIHBO algorithm and its FG variant, FGIHBO, in terms of optimization accuracy, convergence stability, and dimensionality compression capability through multi-dimensional in-depth comparative verification.

The comparative algorithms selected for the experiments encompass 12 intelligent optimization algorithms that have demonstrated outstanding performance in the field of FS in recent years. These include: Multi-strategy Improved Grey Wolf Optimizer (MIGWO) [1], Hybrid Whale Optimization Algorithm based on Gathering strategy (HWOAG) [54], Dynamic Crow Search Algorithm (DCSA) [55], Simulated Annealing (SA) [56], standard Grey Wolf Optimizer (GWO) [57], native Hybrid Breeding Optimization (HBO) [53], Hybrid Breeding Optimization based on Lévy flight and Elite Opposition-Based Learning strategy (LEHBO) [58], alongside its feature grouping variants (CCIHBO2, CCIHBO3, CCIHBO4, CCIHBO5, and CCIHBO6). To ensure the objectivity of the evaluation metrics and effectively suppress the overfitting phenomenon in high-dimensional, small-sample scenarios, all experiments strictly adhere to a standard 5-fold cross-validation procedure.

### 5.1. Experimental Setup and Evaluation Metrics

#### 5.1.1. Benchmark Datasets and Classifier

The simulation experiments in this section utilize 11 gene expression profile datasets sourced from high-dimensional biomedical microarray databases (designated as D1–D11). These datasets exhibit the typical characteristics of small sample sizes and extremely high dimensionality, with feature dimensions ranging from 2000 to 12,533, making them ideal benchmarks for validating the performance of FS algorithms. Table 3 details the biological designations, statistical distributions, and original literature sources of each dataset.

To systematically verify the superiority of the proposed FG strategy, determine the optimal configuration, and simultaneously prevent hyperparameter overfitting on the benchmark suite, this section designs a rigorous two-stage comparative experiment based on a validation-testing separation paradigm:

- Stage 1: Validation of Feature Grouping Mechanism (Datasets D1–D4): To objectively determine the optimal grouping granularity without biasing the final performance evaluation, datasets D1–D4 are strictly utilized as an independent validation set. A comprehensive comparison is conducted among the baseline algorithm MSIHBO, FGIHBO under varying grouping granularities (M∈{2,3,4,5,6}), and LEHBO alongside its grouping variants CCIHBO (M∈{2,3,4,5,6}). This stage identifies the optimal hyperparameter configurations, denoted as BEST and best, respectively.
- Stage 2: Comprehensive Testing in Complex Scenarios (Datasets D5–D11): Utilizing the optimal grouping configurations identified in the validation stage (i.e., $FGIHBO_{M\;=\;BEST}$ and $CCIHBO_{best}$), horizontal testing experiments are performed against the remaining mainstream algorithms on the unseen test datasets D5–D11. This separation ensures that the reported classification accuracy and optimization efficiency on ultra-high-dimensional spaces are generalizable and not the result of dataset-specific hyperparameter tuning.

During the fitness evaluation process, this paper employs the KNN classifier as the criterion function for subset evaluation. To balance classification efficiency and evaluation robustness, the number of nearest neighbors is set to K=5, and the Euclidean Distance is adopted as the metric for sample similarity.

#### 5.1.2. General Experimental Parameter Settings

Regarding the setting of experimental parameters, to ensure fairness and horizontal comparability in evaluating the optimization performance of each algorithm, this section adheres to the principle of combining global uniformity with local specificity. First, for all participating comparative swarm intelligence algorithms, the initial population size *N* is uniformly set to 30, the maximum number of iterations $T_{max}$ is configured to 200, and the heuristic search space is strictly confined within the interval [0,1]. To eliminate the stochastic effects induced by random initialization, all simulation experiments are independently executed 30 times under identical software and hardware environments, with the average values taken as the final statistical results. Furthermore, to ensure a standardized baseline for the subsequent heuristic optimization across datasets with varying scales, the retention dimensionality in the initial mRMR filtering phase is strictly pre-defined. Specifically, the exact retained feature number $D^{\prime }$ for all datasets is uniformly set to 20% of their respective original dimensionality (i.e., $D^{\prime }=0.2\times D$). This unified truncation threshold guarantees that all comparative algorithms are evaluated within the exact same dimensional complexity, thereby ensuring the strict reproducibility of the experiments. Secondly, regarding the algorithm-specific local control parameters inherent to each comparative algorithm, to guarantee a fair comparison and ensure that all competing models are equally tuned to their peak performance, their configurations are strictly set to the optimal values recommended by the original authors in their respective literatures. These specific parameters are detailed in the dedicated parameter tables in Section 5.4 (the grouping mechanism verification stage) and Section 5.5 (the comprehensive performance comparison stage), respectively.

### 5.2. Parameter Sensitivity and Robustness Analysis

In metaheuristic algorithm design, the selection of key control parameters has a direct impact on both optimization performance and convergence stability. To investigate the influence of the core parameters in the proposed FGIHBO framework and to evaluate its robustness under different data environments, a comprehensive one-factor-at-a-time sensitivity analysis was conducted. All experiments were performed on the first four benchmark datasets (D1–D4), using a population size of N=30, a maximum iteration number of $T_{\max}=200$, and a 5-fold cross-validation strategy with the KNN classifier (K=5). Unless otherwise specified, the default parameter settings were fixed as γ=0.90, $D^{\prime }=0.2D$, T=200, and M=5. During the sensitivity analysis, only one parameter was varied at a time while all remaining parameters were kept unchanged. The average classification accuracy over 30 independent runs was adopted as the evaluation criterion.

**Figure 2:**
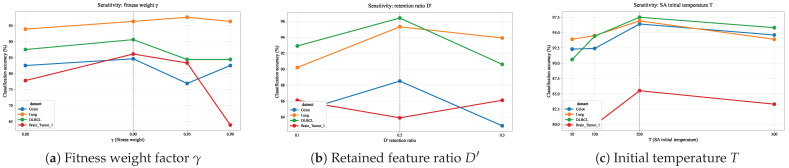
Parameter sensitivity analysis of FGIHBO. (**a**) Sensitivity analysis of the fitness weight factor γ; (**b**) Sensitivity analysis of the retained feature ratio $D^{\prime }$; (**c**) Sensitivity analysis of the simulated annealing initial temperature *T*.

The weighting coefficient γ in the fitness function determines the trade-off between classification accuracy and feature subset compactness. To investigate its influence, γ was varied within the range {0.80,0.90,0.95,0.99}. As illustrated in Figure 2a, the proposed FGIHBO achieves the highest and most stable classification performance across all four datasets when γ=0.90. However, when γ is further increased to 0.95 and 0.99, the classification accuracy on the Brain_Tumor_1 dataset decreases noticeably. This phenomenon suggests that excessively emphasizing classification performance while neglecting feature reduction (i.e., γ approaching 1) may preserve redundant or noisy genes in high-dimensional gene expression datasets, thereby increasing the risk of premature convergence and weakening the generalization ability of the classifier. In contrast, γ=0.90 not only provides the most favorable accuracy gain across multiple datasets but also effectively avoids the performance degradation caused by extreme weighting configurations. Therefore, γ=0.90 is adopted as the default setting in subsequent experiments.

The retained feature ratio $D^{\prime }$ controls the dimensionality of the feature subset after the filtering stage and before the heuristic search process. To determine an appropriate search space size, three candidate ratios were evaluated, namely $D^{\prime }/D\in \left\{0.10,0.20,0.30\right\}$. The results shown in Figure 2b indicate that the proposed method achieves its best overall performance when $D^{\prime }=0.2D$. When the retained ratio is too small ($D^{\prime }=0.1D$), informative genes may be discarded during the filtering phase, resulting in slight underfitting and reduced classification performance. Conversely, when the ratio increases to $D^{\prime }=0.3D$, the enlarged search space introduces additional irrelevant features and increases the computational burden of the optimization process, leading to a moderate decrease in classification accuracy. Therefore, $D^{\prime }=0.2D$ represents an appropriate compromise between information preservation and search efficiency and is selected as the default configuration.

To enhance the capability of escaping local optima, a SA strategy is incorporated into the sterile-line update mechanism. The initial temperature *T* determines the acceptance probability of inferior solutions during the early search stage and therefore directly influences the balance between exploration and exploitation. To evaluate its effect, sensitivity experiments were conducted with T∈{50,100,200,500}. As shown in Figure 2c, all four datasets achieve their highest classification accuracy when T=200, indicating that a moderate initial temperature effectively improves search efficiency while maintaining population diversity. When the temperature is further increased to T=500, the performance of all datasets declines to varying degrees, suggesting that an excessively high acceptance probability weakens the exploitation capability during the later search stages. On the other hand, under relatively low temperatures, the DLBCL and Brain_Tumor_1 datasets exhibit more pronounced performance degradation, implying insufficient exploration and an increased tendency toward premature convergence. Overall, T=200 provides the most effective balance between global exploration and local exploitation and is therefore adopted in all subsequent experiments.

### 5.3. General Optimization Performance Evaluation

To verify the robustness and generalization capability of MSIHBO, the core optimization engine of the proposed FGIHBO framework, on various complex numerical optimization problems, the IEEE CEC2022 benchmark suite was employed for evaluation. The benchmark contains unimodal, multimodal, hybrid, and composition functions, which provide a comprehensive assessment of an algorithm’s global search ability in high-dimensional and highly non-linear landscapes.

The proposed MSIHBO was compared with several representative state-of-the-art metaheuristic algorithms, including Cooperative Multi-population Differential Evolution (CMp-DE) [67], Heavy-tailed Enhanced Human Evolution Optimization (HEST-HEO) [68], Learning Evolution Algorithm (LEA) [69], Particle Swarm Optimization (PSO) [70], Grey Wolf Optimizer (GWO) [57], Hybrid Breeding Optimization (HBO) [53], Learning Elite Hybrid Breeding Optimization (LEHBO) [58], Multi-Strategy Tree Algorithm (MS-TSA) [71], and Improved Carnivorous Plant Algorithm (I-CPA) [72]. To ensure a fair comparison, all algorithm-specific parameters were set according to the recommendations reported in the corresponding original publications. In addition, the population size was fixed at 60, the maximum number of iterations was set to 200, and the problem dimension was set to 10. Each algorithm was independently executed 30 times on every benchmark function. The best, mean, worst, and standard deviation values were recorded for performance evaluation. The detailed experimental results are reported in Table 4, Table 5, Table 6, Table 7, Table 8, Table 9, Table 10, Table 11, Table 12, Table 13, Table 14 and Table 15.

From the mean performance perspective, MSIHBO consistently demonstrated competitive optimization capability across the entire benchmark suite. In particular, on multimodal and composition functions (e.g., F6, F8, and F10), MSIHBO achieved superior solution accuracy compared with the competing algorithms. Moreover, the corresponding standard deviation values remained relatively small, indicating excellent robustness and convergence stability in highly complex search environments.

**Figure 3:**
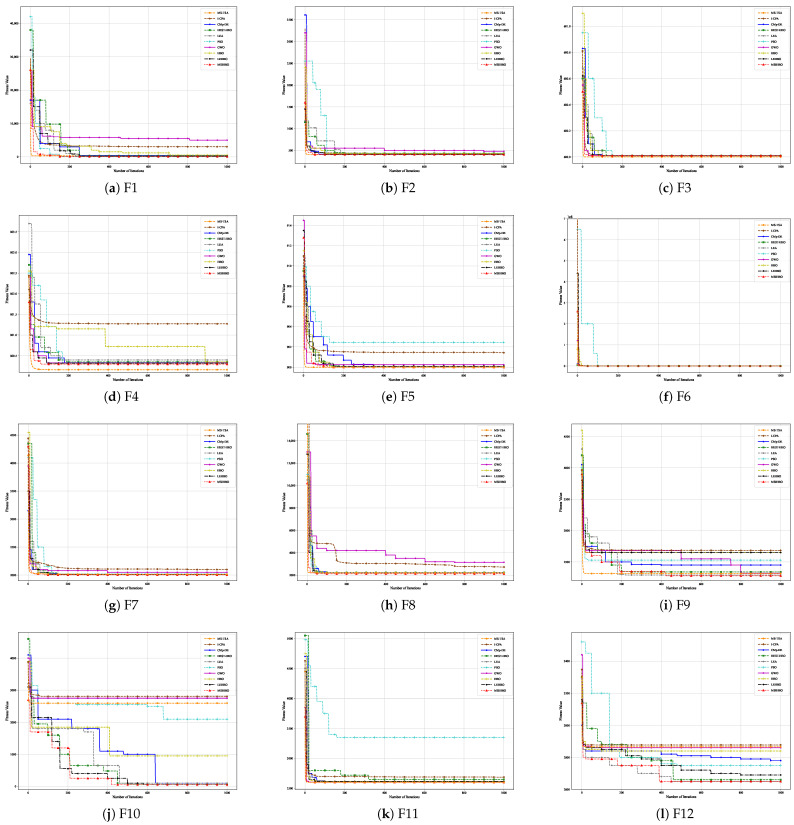
Convergence curves of the compared algorithms on the CEC2022 benchmark functions.

The convergence curves Figure 3 further reveal the search dynamics of the proposed method. Benefiting from the cooperative search mechanism, MSIHBO maintains strong exploration capability during the early evolutionary stages, effectively alleviating premature population aggregation. During the later search process, the synergistic integration of the SA acceptance strategy and the GWO-based guidance mechanism significantly enhances exploitation capability, enabling the algorithm to refine candidate solutions efficiently within promising regions. Consequently, MSIHBO exhibits smoother convergence trajectories and lower terminal errors than the competing methods. Overall, the experimental results demonstrate that MSIHBO is not only an effective optimization engine supporting the FGIHBO framework but also a competitive standalone optimizer for solving complex continuous optimization problems. The superior balance between exploration and exploitation allows the proposed algorithm to achieve high-quality solutions with strong robustness and convergence reliability across different categories of benchmark functions.

**Figure 4:**
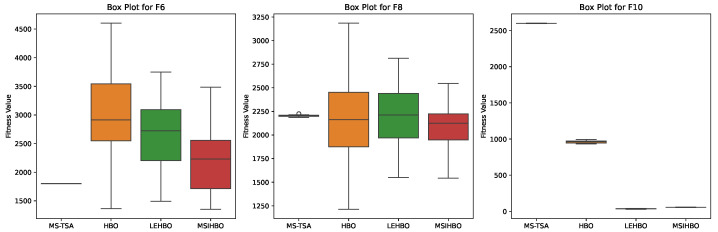
Box plot comparison of MSIHBO, MS-TSA, and LEHBO on representative CEC2022 benchmark functions (F6, F8, and F10) over 30 independent runs.

The box plots in Figure 4 visually summarize the distribution of results over 30 independent runs. The narrower interquartile ranges and lower median fitness values achieved by MSIHBO across functions F6, F8, and F10 demonstrate its superior consistency and stability in navigating complex search landscapes compared to existing robust algorithms like MS-TSA and LEHBO.

**Figure 5:**
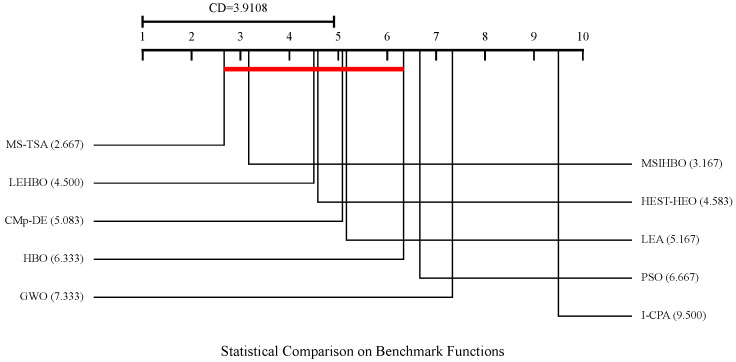
CD diagram obtained from the Friedman and Nemenyi statistical tests on the CEC2022 benchmark functions. Lower average ranks indicate better optimization performance. Algorithms connected by the red horizontal line are not significantly different at the 0.05 significance level.

To further investigate the statistical significance of the performance differences, the Friedman test combined with the Nemenyi post-hoc analysis was conducted on the twelve CEC2022 benchmark functions. The average ranking results are presented in Figure 5. MS-TSA achieved the best average rank (2.667), followed by the proposed MSIHBO (3.167). The calculated critical difference (CD) at the 0.05 significance level was 3.9108. Since the rank difference between MS-TSA and MSIHBO was smaller than the CD value, no statistically significant difference was observed between the two algorithms. Nevertheless, both methods obtained substantially better rankings than most competing algorithms, demonstrating the strong optimization capability and robustness of the proposed MSIHBO across diverse benchmark problems.

### 5.4. Verification of Feature Grouping Mechanism and Optimization of Parameter Granularity

In high-dimensional FS tasks, the grouping granularity (i.e., the number of feature subspaces *M*) directly determines the degree of decoupling of the high-dimensional feature space. If *M* is set too small, the feature space is not sufficiently reduced, making it difficult to effectively mitigate the curse of dimensionality; conversely, if *M* is set too large, an overly fragmented partitioning will not only disrupt strongly correlated feature combinations, but also lead to a severe lack of genetic diversity within the resulting subpopulations, increasing the risk of entrapment in local optima.

To investigate the specific impact of grouping granularity on the optimization performance of the algorithms and to determine the optimal parameter configuration for the proposed method, this section conducts a grouping strategy optimization experiment on four high-dimensional biomedical datasets, D1–D4. The specific internal parameter settings for the various comparative algorithms participating in this stage are detailed in Table 16. The experiment evaluated five metrics for MSIHBO, LEHBO, and their synergistic evolutionary variants under different grouping scales (M∈{2,3,4,5,6}), denoted as $FGIHBO_{M\;=\;2∼6}$ and $CCIHBO_{M\;=\;2∼6}$: Best fitness (Best), Worst fitness (Worst), Mean fitness (Mean), Standard Deviation (Std), and execution Time (Time).

The experimental results, as illustrated in Table 17, demonstrate that the proposed MSIHBO algorithm outperforms the baseline algorithm LEHBO across all performance metrics on the four datasets. Taking dataset D1 as an example, MSIHBO not only achieves higher fitness but also exhibits a smaller standard deviation, demonstrating stronger optimization stability. On the higher-dimensional datasets D3 and D4, MSIHBO continues to maintain superior performance, validating the effectiveness of the multi-strategy synergistic mechanism within complex feature spaces.

Following the introduction of the FG strategy, the performance of the FGIHBO series algorithms is further enhanced. Their Best, Worst, and Mean fitness values across all datasets (D1–D4) are superior to the ungrouped MSIHBO, fully validating the positive role of the grouping strategy in improving global search capabilities. In contrast, the other comparative group, the CCIHBO series, fails to comprehensively surpass the original LEHBO, with some variants even exhibiting a decline in the optimal fitness. This indicates that traditional synergistic evolutionary grouping strategies remain insufficiently stable in FS tasks. Although FGIHBO experiences a marginal increase in execution time and standard deviation, the substantial improvement in solution quality is of paramount importance.

Under identical grouping quantity conditions (e.g., M=5), the overall performance of FGIHBO similarly outperforms that of CCIHBO. Notably, on the high-dimensional dataset D4, the optimal fitness of $FGIHBO_{M\;=\;5}$ reaches 97.16%, surpassing the counterpart CCIHBO5 (84.04%) by 13.12%, further substantiating the superiority of the proposed grouping strategy in high-dimensional scenarios. Moreover, facing the significant escalation in dimensionality from D3 to D4, FGIHBO exhibits no performance degradation, demonstrating robust adaptability to dimensional variations.

Synthesizing all evaluation metrics, $FGIHBO_{M\;=\;5}$ and CCIHBO3 are identified as the optimal configurations within their respective series. Since fitness (classification accuracy) serves as the most critical evaluation criterion in FS tasks, $FGIHBO_{M\;=\;5}$ can be regarded as the algorithm with the best comprehensive performance in this stage.

**Figure 6:**
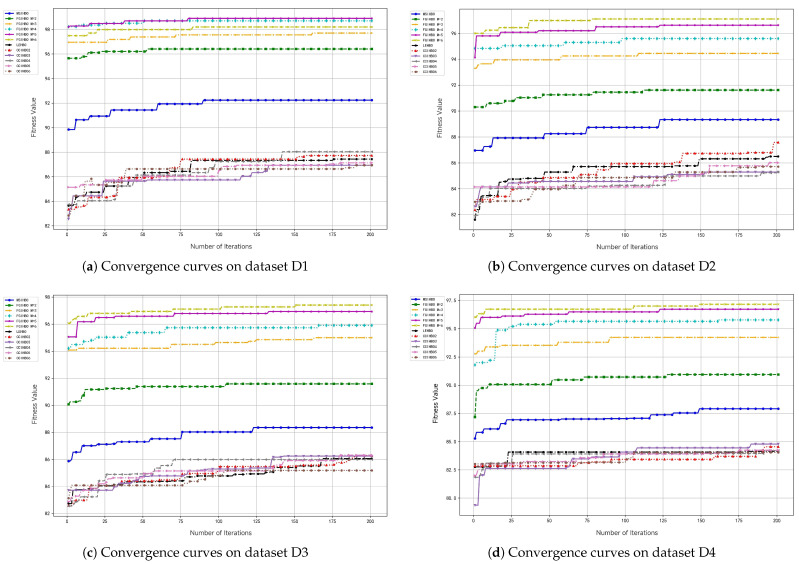
Experimental results of convergence curves across datasets D1–D4.

To further analyze the dynamic optimization characteristics of the participating comparative algorithms, Figure 6 illustrates their accuracy convergence curves across the different datasets. For the baseline algorithms without grouping, MSIHBO exhibits a convergence advantage over LEHBO and the CCIHBO series throughout the entire evolutionary process. MSIHBO demonstrates a higher initial starting point and a steeper early convergence trend, indicating its capability to rapidly lock onto superior search regions. During the middle and late stages of iteration, its curve exhibits minor fluctuations and achieves a higher final fitness, verifying the dual effects of the multi-strategy synergistic mechanism in accelerating convergence and maintaining search stability.

In contrast, the FGIHBO series algorithms, which incorporate the FG strategy, open up a significant performance gap with the other comparative models. As is evident from the figures, the FGIHBO series not only converges extremely fast in the initial stages but also achieves final convergence values that far exceed those of other algorithms. This global lead is primarily attributed to the divide-and-conquer dimensionality reduction and decoupling mechanism: by partitioning the high-dimensional features into multiple subspaces for parallel optimization, the algorithm effectively circumvents the curse of dimensionality, thereby significantly enhancing the synergistic evolutionary efficiency among individuals while simultaneously boosting search diversity.

Furthermore, a horizontal comparison regarding the impact of grouping granularity (the *M* value) reveals that on medium-to-high-dimensional datasets such as D2 and D3, the overall convergence accuracy of FGIHBO presents an increasing trend with the augmentation of the number of groups. This demonstrates that finer-grained feature partitioning facilitates the capture of crucial gene combinations. Conversely, the performance of the traditional CCIHBO series fluctuates drastically as the number of groups increases, failing to exhibit a clear positive correlation trend. This phenomenon further indirectly corroborates that the FG method proposed in this paper possesses superior robustness and scalability when processing complex high-dimensional data.

### 5.5. Comprehensive Performance Comparative Analysis in Complex High-Dimensional Scenarios

To verify the comprehensive performance of the FGIHBO method when processing large-scale feature spaces, this section selects the optimal configurations determined in the first stage, namely $FGIHBO_{M\;=\;5}$ and CCIHBO3, and conducts a comprehensive comparison against seven intelligent optimization algorithms (MIGWO, HWOAG, DCSA, SA, GWO, HBO, and LEHBO) on seven biomedical datasets with broader dimensions (D5–D11). The core control parameters for each comparative algorithm are set according to the recommended values in their original papers; specific parameter configurations are detailed in Table 18. The experimental results of each comparative algorithm are presented in Table 19, providing an in-depth evaluation of each method’s optimization accuracy and robustness when confronted with exponential expansion in feature space dimensionality.

To explicitly quantify the performance superiority of the proposed framework, the absolute improvement in the average classification accuracy is utilized as the primary evaluation metric in our comparative analysis. The calculation formula is defined as follows:

(24) $$\mathrm{Improvement}\left(\%\right)={\bar{Acc}}_{FGIHBO}-{\bar{Acc}}_{baseline}$$

where ${\bar{Acc}}_{FGIHBO}$ represents the average classification accuracy achieved by the proposed FGIHBO across 30 independent runs, and ${\bar{Acc}}_{baseline}$ represents the average accuracy of a specific baseline algorithm under identical experimental conditions.

The experimental results demonstrate that across the D5–D11 test datasets, which feature significant dimensional leaps, the Best, Worst, and Mean fitness of the baseline MSIHBO algorithm comprehensively surpass those of HBO and LEHBO, and are significantly superior to other comparative algorithms such as SA and MIGWO. This verifies the effectiveness of the multi-strategy synergistic mechanism within high-dimensional search spaces. Building upon this foundation, the introduction of the grouping strategy in $FGIHBO_{M\;=\;5}$ further widens the performance gap. Compared to the similar grouping algorithm CCIHBO3, $FGIHBO_{M\;=\;5}$ maintains a comprehensive lead across all metrics, proving that the FG mechanism proposed in this paper is more rational and efficient in subspace cooperative searching. Regarding the standard deviation metric, although $FGIHBO_{M\;=\;5}$ theoretically exhibits extremely slight fluctuations on certain ultra-high-dimensional datasets, its average fitness consistently remains at the highest level across the board; thus, these minor fluctuations do not substantively undermine the quality of the final solution. In summary, in extreme high-dimensional scenarios, the proposed method perfectly balances the breakthrough of the upper limit of classification accuracy with the stability of the solution.

**Figure 7:**
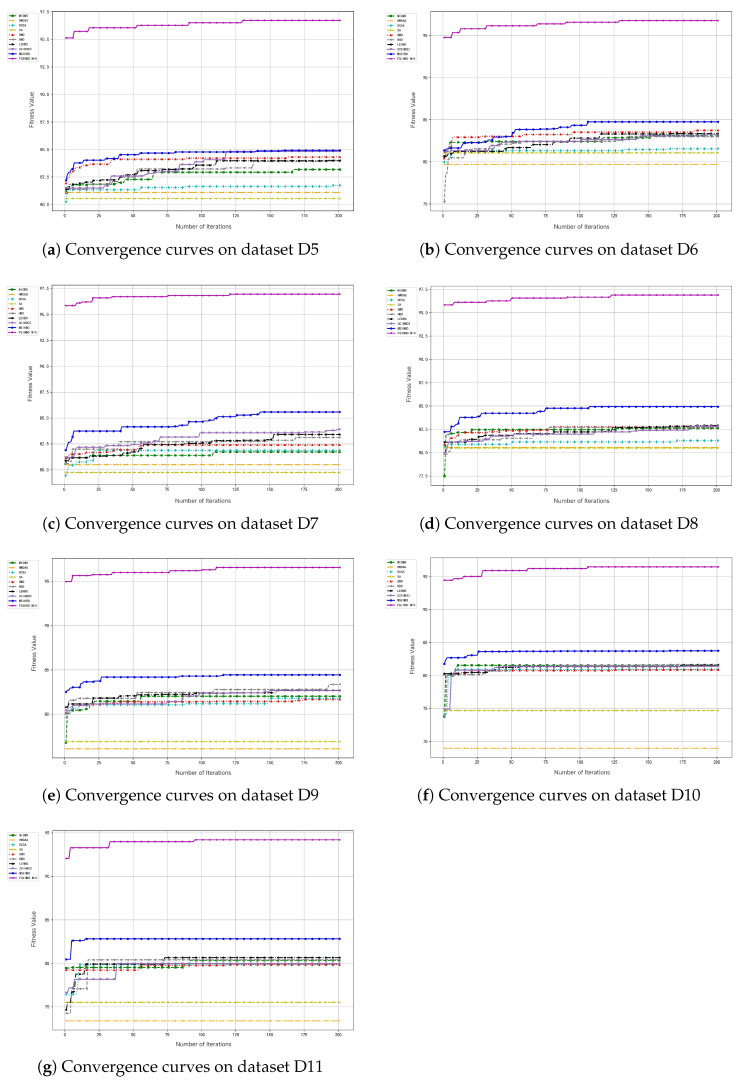
Experimental results of convergence curves across datasets D5–D11.

Figure 7 illustrates the convergence curves of the algorithms on datasets D5–D11. As observed from the figures, the convergence process of MSIHBO exhibits a typical step-like elevation characteristic. Compared to the original HBO, it possesses a faster convergence rate and a higher final fitness, demonstrating excellent resistance to premature convergence. This indicates that the improvements introduced to HBO in MSIHBO are effective at the search mechanism level. Upon integrating the grouping strategy, the convergence performance of $FGIHBO_{M\;=\;5}$ achieves a further leap. It not only possesses a higher initial search starting point but also continuously breaks through stagnation and escapes local optima during the middle and late stages of iteration. This capability to consistently and rapidly approximate the global optimal solution within ultra-high-dimensional spaces fully demonstrates that the proposed grouping strategy possesses exceptional scalability. Even when the feature dimensionality reaches the tens of thousands, it can still efficiently lock onto the crucial feature subsets. The final convergence accuracy establishes a significant gap compared to other algorithms. This indicates that the FG mechanism vastly enhances the parallel exploration capability of the algorithm in high-dimensional spaces, enabling it to swiftly approximate the global optimum within a smaller number of iterations.

**Figure 8:**
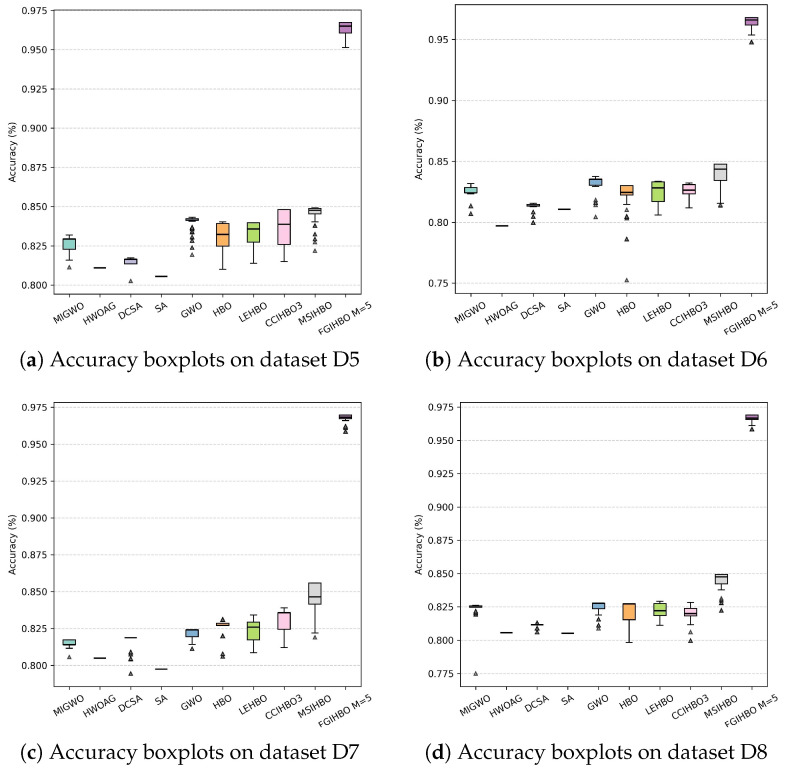

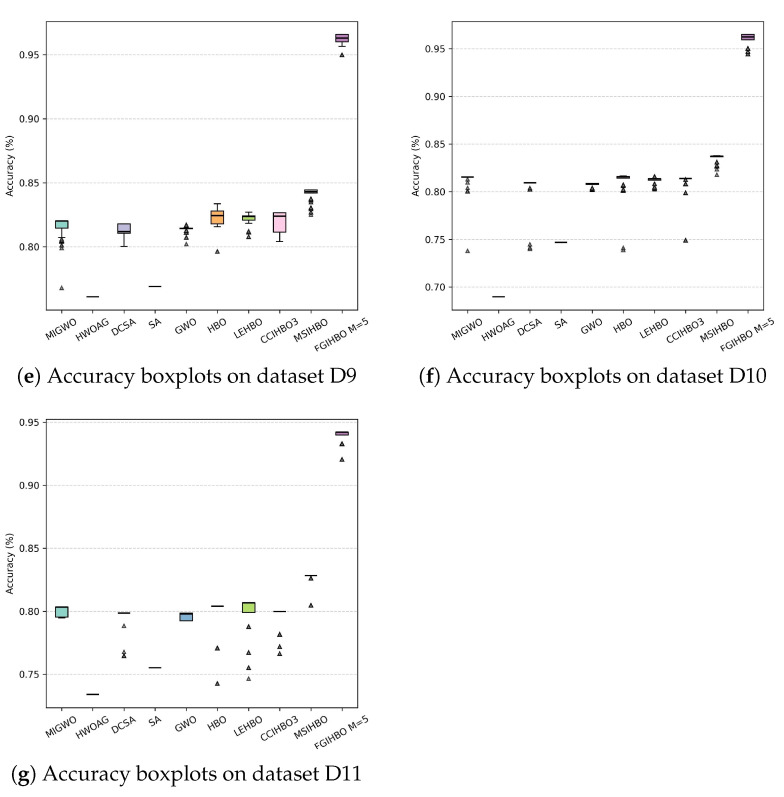
Experimental results of accuracy boxplots across datasets D5–D11.

To intuitively reflect the fluctuation of the algorithms across multiple independent runs, Figure 8 plots the accuracy boxplots for each comparative algorithm. The experiments reveal that the accuracy distribution range of $FGIHBO_{M\;=\;5}$ is the narrowest, with shorter boxes and medians consistently maintained at the highest level, exhibiting extremely strong robustness and stable convergence capabilities. In contrast, other traditional or improved algorithms exhibit varying degrees of limitations in stability. For instance, although HWOAG and SA have relatively narrow boxes, they present numerous outliers with large spans, reflecting frequent premature convergence phenomena. The noticeably long boxes and whiskers of DCSA and HBO indicate that their performance fluctuates drastically depending on initialization. While the results for LEHBO and CCIHBO3 are relatively concentrated, they are still accompanied by a certain number of outliers in complex high-dimensional problems. Overall, $FGIHBO_{M\;=\;5}$ is the only algorithm in the test capable of consistently and stably outputting high-quality feature subsets.

### 5.6. Ablation Study

To verify the effectiveness of the key components incorporated into the proposed FGIHBO framework, including the mRMR-based pre-filtering strategy, dynamic FG mechanism, high-quality population initialization scheme, and multi-strategy cooperative optimization engine, comprehensive ablation experiments were conducted on four high-dimensional biomedical datasets (D1–D4).

The complete framework with the optimal grouping granularity (M=5), denoted as FGIHBO5, was regarded as the baseline model. Six variant models were constructed for comparison:

1. MSIHBO: the FG mechanism was completely removed, reducing the search process to a direct optimization in the original feature space.
2. FGIHBO5 w/o mRMR: the mRMR pre-filtering stage was discarded to evaluate the importance of preliminary redundant-feature elimination.
3. FGIHBO5 w/o LHS+EOBL: LHS and EOBL were removed and replaced by random initialization.
4. FGIHBO5 w/o DynamicPartition: the roulette-wheel-based dynamic partition mechanism was removed to assess the effectiveness of adaptive feature-space decomposition.
5. FGIHBO5 w/o GWO: the GWO-guided maintainer update strategy was excluded to evaluate its contribution to exploitation capability.
6. FGIHBO5 w/o SA: the SA acceptance mechanism and entropy-based cooling strategy were removed to investigate their role in maintaining population diversity and escaping local optima.

**Figure 9:**
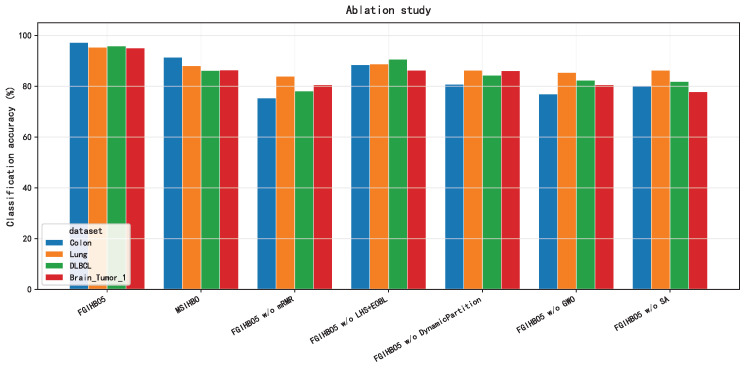
Classification accuracies of the complete FGIHBO framework and its ablation variants on the four benchmark datasets.

Each variant was independently executed 30 times on every dataset, and the average classification accuracy was recorded. Table 20 summarizes the structural differences among the ablation variants. The corresponding classification performances are reported in Table 21, while Figure 9 provides a visual comparison of their relative contributions across the four benchmark datasets.

As observed from Table 21, the complete FGIHBO5 framework consistently achieved the highest classification accuracy across all datasets, demonstrating the effectiveness and complementarity of its constituent modules.

The contribution of the dimensionality-reduction mechanism is particularly evident. Removing the mRMR pre-filtering stage resulted in the most severe performance degradation, with the classification accuracy on D1 dropping from 97.25% to 75.38%. This substantial decline confirms that eliminating highly redundant features prior to evolutionary search is crucial for reducing search complexity and improving optimization efficiency in high-dimensional feature spaces. Similarly, both the removal of feature grouping (MSIHBO) and the elimination of the dynamic partition strategy led to noticeable performance deterioration on all datasets, indicating that adaptive feature-space decomposition effectively alleviates the curse of dimensionality and captures latent feature interactions.

The population initialization strategy also plays an important role in maintaining search quality. When LHS and EOBL were removed, classification accuracy decreased by approximately 4–9% across the datasets, suggesting that high-quality initial solutions significantly enhance population diversity and facilitate global exploration during the early search stage.

Furthermore, both the GWO-guided exploitation mechanism and the SA-based diversification strategy were shown to be indispensable. Excluding either component caused substantial performance losses. In particular, removing the SA mechanism reduced the accuracy on D4 to 77.78%, implying that the entropy-guided simulated annealing acceptance mechanism effectively maintains population diversity during the early search stage and enhances local exploitation capability in the later stage, thereby preventing premature convergence and improving the algorithm’s ability to escape local optima.

Overall, the ablation results demonstrate that each module contributes positively to the final optimization performance. The superior accuracy achieved by the complete FGIHBO5 framework confirms that the proposed components cooperate synergistically, jointly enhancing both exploration and exploitation capabilities for high-dimensional biomedical FS tasks.

### 5.7. Statistical Significance Analysis

To rigorously assess whether the performance superiority of the proposed FGIHBO framework over competing algorithms is statistically significant, a non-parametric multiple comparison procedure was conducted. Specifically, the Friedman test was first employed to evaluate the overall ranking differences among algorithms, followed by the Nemenyi post-hoc test to identify pairwise statistical significance.

**Figure 10:**
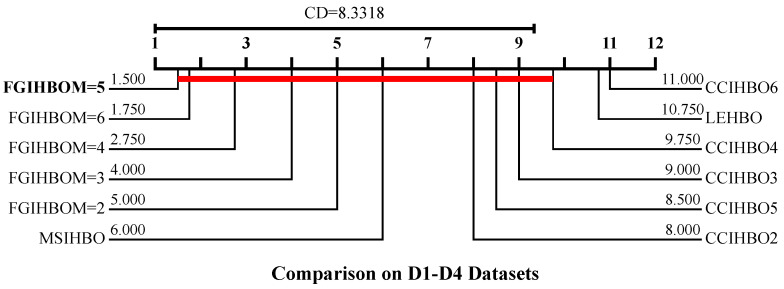
CD diagram of the compared algorithms on datasets D1–D4 based on the Friedman and Nemenyi tests. Algorithms connected by the red line are not significantly different at the 0.05 significance level.

**Figure 11:**
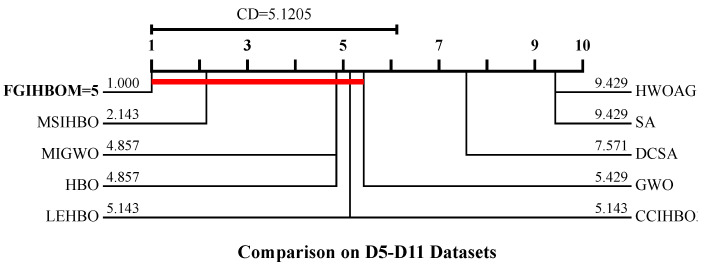
CD diagram of the compared algorithms on datasets D5–D11 based on the Friedman and Nemenyi tests. Algorithms connected by the red line are not significantly different at the 0.05 significance level.

Figure 10 and Figure 11 present the CD diagrams obtained from the Nemenyi post-hoc analysis for the D1–D4 and D5–D11 benchmark datasets, respectively. In the first-stage experiments involving datasets D1–D4, the complete FGIHBO framework (denoted as FGIHBO_*M* = 5_) achieved the best average rank of 1.500, outperforming all competing algorithms. Although the corresponding critical difference threshold was relatively large (CD=8.3318) due to the limited number of datasets, FGIHBO_*M* = 5_ still demonstrated statistically superior ranking performance compared with several lower-ranked competitors, including LEHBO and CCIHBO6.

In the second-stage experiments conducted on the more challenging high-dimensional datasets D5–D11, FGIHBO_*M* = 5_ further exhibited remarkable optimization capability and achieved the optimal average rank of 1.000. According to the Nemenyi test, the calculated critical difference value was CD=5.1205. Under this stricter evaluation setting, FGIHBO_*M* = 5_ not only consistently outperformed its underlying optimizer MSIHBO (average rank = 2.143), but also achieved statistically significant superiority over several representative metaheuristic algorithms, including HWOAG, SA, and DCSA, whose average ranks fell outside the corresponding significance boundaries in the CD diagram.

Overall, the Friedman and Nemenyi statistical analyses consistently verify that the proposed FGIHBO framework achieves superior ranking performance across datasets with varying dimensional characteristics. These findings provide strong statistical evidence that the introduced dynamic feature partitioning strategy and multi-strategy collaborative optimization mechanism effectively and reliably enhance search performance for high-dimensional feature selection problems.

## 6. Conclusions

To address the curse of dimensionality and optimization stagnation issues frequently encountered in FS for high-dimensional biomedical data, this paper proposes a dimensionality reduction method based on FG and Improved HBO. The study achieves a low-coupling reconstruction of the high-dimensional search space through the mRMR and SU criteria. Furthermore, it innovatively integrates the encircling guidance strategy of the GWO and an adaptive annealing mechanism based on Shannon Entropy, thereby accomplishing a multi-strategy upgrade of the underlying evolutionary optimizer. Systematic validation across datasets D1–D11 demonstrates that the method effectively circumvents the computational bottleneck induced by the exponential expansion of the feature space. It exhibits formidable scalability and robustness while ensuring exceptionally high classification accuracy. Despite these advantages, the proposed method relies on a KNN-based wrapper evaluation paradigm, which still encounters a computational bottleneck when processing ultra-large-scale datasets. Therefore, future research will primarily focus on three directions to extend this work. First, to alleviate the computational burden, we intend to introduce a Filter-Wrapper hybrid architecture or surrogate-assisted evaluation models to partially replace expensive exact evaluations. Second, to eliminate the reliance on the empirical weight parameter in the fitness function, we plan to develop a multi-objective variant based on Pareto dominance, providing decision-makers with a diverse set of non-dominated optimal trade-off solutions. Finally, we will explore the adaptability of the SU-based bucket grouping mechanism in other complex domains, such as unstructured image and text mining, where intrinsic spatial or semantic correlations can be further exploited.

## Figures and Tables

**Table 1:** Qualitative comparison of representative feature grouping and feature selection methods reported in the literature.

Category	Methodology	Key Results/ Strengths	Core Limitations
Linear Correlation Grouping [29]	Pearson/ distance-based linear correlation metrics.	Low computational overhead; isolates explicit collinearity.	Misses complex non-linear interactions; sensitive to feature scales.
Information-Theoretic Grouping [31,32,33,34,35]	Non-linear entropy measures (MI, SU).	Captures non-linear associations; scale-invariant evaluation.	Suffers from empirical estimation bias; lacks spatial search synergy.
Clustering-based Partitioning [38,40,41]	Unsupervised feature clustering (*k*-means, community detection).	Label-free division; enhances population diversity.	High sensitivity to cluster initialization; breaks strong couplings.
Standard Ranking Partitioning [39,42]	Deterministic division based on relevance scores.	Simplifies search space; low computational complexity.	Lacks strict normalization; heavily sequence-dependent.
Traditional Metaheuristics [45,46,47,48]	Full-space continuous search via binary wrappers (GA, PSO, GWO).	Robust global search; gradient-free optimization.	Suffers from the curse of dimensionality; late-stage local stagnation.

**Table 2:** Component-wise comparison of the core mechanisms between the standard HBO and the proposed MSIHBO.

Algorithmic Component	Standard HBO	MSIHBO
Search space	High-dimensional space	Modular subspaces via SU-based grouping
Population initialization	Random initialization	LHS with EOBL
Partitioning strategy	Static ranking	Dynamic roulette wheel selection
Maintainer line	No position update	GWO-guided exploitation with non-linear cosine decay
Restorer line	Standard selfing	Standard selfing
Sterile line	Greedy replacement	SA with Shannon entropy-driven temperature

**Table 3:** Summary of high-dimensional biomedical microarray datasets.

ID	Dataset	Samples	Features	Classes	Ref.
D1	Colon Cancer (Colon)	62	2000	2	[59]
D2	Lung Carcinoma (Lung)	203	3312	5	[60]
D3	Diffuse Large B-Cell Lymphoma (DLBCL)	77	5469	2	[61]
D4	Brain Tumor Phase I (Brain_Tumor_1)	90	5920	5	[62]
D5	Lymphoma	96	4026	9	[63]
D6	GLIOMA	50	4434	4	[64]
D7	Leukemia Phase I (Leukemia_1)	72	5327	3	[65]
D8	Acute Leukemia (ALLAML)	72	7129	2	[65]
D9	Leukemia Phase II (Leukemia_2)	72	7129	4	[65]
D10	Brain Tumor Phase II (Brain_Tumor_2)	50	10,367	4	[64]
D11	11-Tumor	174	12,533	11	[66]

**Table 4:** Experimental results on CEC2022 benchmark function F1.

Algorithm	Best	Mean	Worst	Std
MS-TSA [71]	**3.000 × 10^2^**	**3.000 × 10^2^**	**3.000 × 10^2^**	**5.684 × 10^−14^**
I-CPA [72]	3.000 × 10^2^	3.015 × 10^3^	3.240 × 10^4^	6.869 × 10^3^
CMp-DE [67]	3.010 × 10^2^	3.066 × 10^2^	3.150 × 10^2^	9.899
HEST-HEO [68]	3.002 × 10^2^	3.020 × 10^2^	4.000 × 10^2^	6.930 × 10^2^
LEA [69]	3.037 × 10^2^	3.069 × 10^2^	3.162 × 10^2^	6.576
PSO [70]	3.200 × 10^2^	3.237 × 10^2^	3.940 × 10^2^	4.971 × 10^1^
GWO [57]	4.501 × 10^3^	5.040 × 10^3^	5.340 × 10^3^	2.121 × 10^2^
HBO [53]	5.003 × 10^2^	5.432 × 10^2^	6.110 × 10^2^	4.794 × 10^1^
LEHBO [58]	**3.000 × 10^2^**	**3.000 × 10^2^**	3.205 × 10^2^	1.450 × 10^1^
MSIHBO	**3.000 × 10^2^**	**3.000 × 10^2^**	3.003 × 10^2^	2.121 × 10^−1^

**Table 5:** Experimental results on CEC2022 benchmark function F2.

Algorithm	Best	Mean	Worst	Std
MS-TSA [71]	**4.000 × $10^{2}$**	4.035 × $10^{2}$	4.089 × $10^{2}$	3.222
I-CPA [72]	**4.000 × $10^{2}$**	4.230 × $10^{2}$	6.703 × $10^{2}$	4.283 × $10^{1}$
CMp-DE [67]	4.005 × $10^{2}$	4.075 × $10^{2}$	4.250 × $10^{2}$	1.732 × $10^{1}$
HEST-HEO [68]	4.023 × $10^{2}$	4.054 × $10^{2}$	4.354 × $10^{2}$	2.121 × $10^{1}$
LEA [69]	4.200 × $10^{2}$	4.229 × $10^{2}$	4.415 × $10^{2}$	1.315 × $10^{1}$
PSO [70]	**4.000 × $10^{2}$**	**4.000 × $10^{2}$**	**4.009 × $10^{2}$**	**6.364 × $10^{-1}$**
GWO [57]	4.457 × $10^{2}$	4.854 × $10^{2}$	5.053 × $10^{2}$	1.407 × $10^{1}$
HBO [53]	4.027 × $10^{2}$	4.089 × $10^{2}$	4.407 × $10^{2}$	2.249 × $10^{1}$
LEHBO [58]	4.054 × $10^{2}$	4.088 × $10^{2}$	4.189 × $10^{2}$	7.142
MSIHBO	**4.000 × $10^{2}$**	4.058 × $10^{2}$	4.108 × $10^{2}$	3.536

**Table 6:** Experimental results on CEC2022 benchmark function F3.

Algorithm	Best	Mean	Worst	Std
MS-TSA [71]	**6.000 × $10^{2}$**	**6.000 × $10^{2}$**	**6.000 × $10^{2}$**	**2.849 × $10^{-13}$**
I-CPA [72]	**6.000 × $10^{2}$**	6.001 × $10^{2}$	6.001 × $10^{2}$	2.324 × $10^{-2}$
CMp-DE [67]	**6.000 × $10^{2}$**	**6.000 × $10^{2}$**	6.001 × $10^{2}$	7.671 × $10^{-2}$
HEST-HEO [68]	6.010 × $10^{2}$	6.013 × $10^{2}$	6.025 × $10^{2}$	8.485 × $10^{-1}$
LEA [69]	6.002 × $10^{2}$	6.002 × $10^{2}$	6.012 × $10^{2}$	7.071 × $10^{-1}$
PSO [70]	**6.000 × $10^{2}$**	6.001 × $10^{2}$	6.003 × $10^{2}$	1.414 × $10^{-1}$
GWO [57]	6.001 × $10^{2}$	6.002 × $10^{2}$	6.004 × $10^{2}$	1.414 × $10^{-1}$
HBO [53]	**6.000 × $10^{2}$**	**6.000 × $10^{2}$**	6.004 × $10^{2}$	2.828 × $10^{-1}$
LEHBO [58]	**6.000 × $10^{2}$**	**6.000 × $10^{2}$**	6.002 × $10^{2}$	1.414 × $10^{-1}$
MSIHBO	**6.000 × $10^{2}$**	**6.000 × $10^{2}$**	6.001 × $10^{2}$	7.071 × $10^{-2}$

**Table 7:** Experimental results on CEC2022 benchmark function F4.

Algorithm	Best	Mean	Worst	Std
MS-TSA [71]	8.001 × $10^{2}$	8.002 × $10^{2}$	8.003 × $10^{2}$	5.235 × $10^{-2}$
I-CPA [72]	8.001 × $10^{2}$	8.013 × $10^{2}$	8.027 × $10^{2}$	7.851 × $10^{-1}$
CMp-DE [67]	8.0024 × $10^{2}$	8.0034 × $10^{2}$	8.0044 × $10^{2}$	1.4722 × $10^{-1}$
HEST-HEO [68]	8.0030 × $10^{2}$	8.0036 × $10^{2}$	8.0041 × $10^{2}$	3.5355 × $10^{-2}$
LEA [69]	8.0029 × $10^{2}$	8.0030 × $10^{2}$	8.0034 × $10^{2}$	2.8284 × $10^{-2}$
PSO [70]	8.0030 × $10^{2}$	8.0035 × $10^{2}$	8.0039 × $10^{2}$	2.8284 × $10^{-2}$
GWO [57]	8.0027 × $10^{2}$	8.0030 × $10^{2}$	8.0033 × $10^{2}$	2.1213 × $10^{-2}$
HBO [53]	8.0025 × $10^{2}$	8.0030 × $10^{2}$	8.0035 × $10^{2}$	3.5355 × $10^{-2}$
LEHBO [58]	8.0024 × $10^{2}$	8.0031 × $10^{2}$	8.0033 × $10^{2}$	1.4754 × $10^{-2}$
MSIHBO	**8.0020 × $10^{2}$**	**8.0025 × $10^{2}$**	**8.0027 × $10^{2}$**	**1.4142 × $10^{-2}$**

**Table 8:** Experimental results on CEC2022 benchmark function F5.

Algorithm	Best	Mean	Worst	Std
MS-TSA [71]	**9.000 × $10^{2}$**	**9.000 × $10^{2}$**	**9.001 × $10^{2}$**	**2.429 × $10^{-2}$**
I-CPA [72]	**9.000 × $10^{2}$**	9.014 × $10^{2}$	9.091 × $10^{2}$	1.926
CMp-DE [67]	9.011 × $10^{2}$	9.030 × $10^{2}$	9.051 × $10^{2}$	2.828
HEST-HEO [68]	**9.000 × $10^{2}$**	9.002 × $10^{2}$	9.201 × $10^{2}$	1.407 × $10^{1}$
LEA [69]	9.001 × $10^{2}$	9.002 × $10^{2}$	9.011 × $10^{2}$	6.364 × $10^{-1}$
PSO [70]	9.005 × $10^{2}$	9.025 × $10^{2}$	9.032 × $10^{2}$	4.950 × $10^{-1}$
GWO [57]	9.002 × $10^{2}$	9.003 × $10^{2}$	9.013 × $10^{2}$	7.071 × $10^{-1}$
HBO [53]	9.001 × $10^{2}$	9.003 × $10^{2}$	9.015 × $10^{2}$	8.485 × $10^{-1}$
LEHBO [58]	**9.000 × $10^{2}$**	9.001 × $10^{2}$	9.011 × $10^{2}$	7.071 × $10^{-1}$
MSIHBO	**9.000 × $10^{2}$**	**9.000 × $10^{2}$**	9.006 × $10^{2}$	4.243 × $10^{-1}$

**Table 9:** Experimental results on CEC2022 benchmark function F6.

Algorithm	Best	Mean	Worst	Std
MS-TSA [71]	**1.800 × $10^{3}$**	**1.801 × $10^{3}$**	**1.806 × $10^{3}$**	**9.829 × $10^{-1}$**
I-CPA [72]	8.067 × $10^{3}$	4.732 × $10^{4}$	1.012 × $10^{5}$	2.654 × $10^{4}$
CMp-DE [67]	8.232 × $10^{3}$	1.013 × $10^{4}$	1.935 × $10^{4}$	7.862 × $10^{3}$
HEST-HEO [68]	5.014 × $10^{3}$	6.925 × $10^{3}$	7.757 × $10^{3}$	5.883 × $10^{2}$
LEA [69]	6.724 × $10^{3}$	8.108 × $10^{3}$	8.963 × $10^{3}$	6.046 × $10^{2}$
PSO [70]	9.502 × $10^{3}$	1.013 × $10^{4}$	1.591 × $10^{4}$	4.087 × $10^{3}$
GWO [57]	2.503 × $10^{4}$	3.492 × $10^{4}$	4.547 × $10^{4}$	7.460 × $10^{3}$
HBO [53]	2.341 × $10^{3}$	3.108 × $10^{3}$	4.336 × $10^{3}$	8.683 × $10^{2}$
LEHBO [58]	1.869 × $10^{3}$	2.660 × $10^{3}$	3.562 × $10^{3}$	6.378 × $10^{2}$
MSIHBO	1.869 × $10^{3}$	2.200 × $10^{3}$	2.996 × $10^{3}$	5.629 × $10^{2}$

**Table 10:** Experimental results on CEC2022 benchmark function F7.

Algorithm	Best	Mean	Worst	Std
MS-TSA [71]	2.000 × $10^{3}$	2.014 × $10^{3}$	**2.022 × $10^{3}$**	**8.727**
I-CPA [72]	2.024 × $10^{3}$	2.099 × $10^{3}$	2.339 × $10^{3}$	6.945 × $10^{1}$
CMp-DE [67]	1.356 × $10^{3}$	**2.011 × $10^{3}$**	2.596 × $10^{3}$	8.768 × $10^{2}$
HEST-HEO [68]	1.421 × $10^{3}$	2.015 × $10^{3}$	2.634 × $10^{3}$	4.377 × $10^{2}$
LEA [69]	1.381 × $10^{3}$	2.040 × $10^{3}$	2.515 × $10^{3}$	3.359 × $10^{2}$
PSO [70]	1.702 × $10^{3}$	2.029 × $10^{3}$	2.503 × $10^{3}$	3.352 × $10^{2}$
GWO [57]	1.450 × $10^{3}$	2.055 × $10^{3}$	2.639 × $10^{3}$	4.130 × $10^{2}$
HBO [53]	1.331 × $10^{3}$	2.031 × $10^{3}$	2.579 × $10^{3}$	3.875 × $10^{2}$
LEHBO [58]	1.624 × $10^{3}$	2.024 × $10^{3}$	2.604 × $10^{3}$	4.101 × $10^{2}$
MSIHBO	**1.237 × $10^{3}$**	2.027 × $10^{3}$	2.431 × $10^{3}$	2.998 × $10^{2}$

**Table 11:** Experimental results on CEC2022 benchmark function F8.

Algorithm	Best	Mean	B	Std
MS-TSA [71]	2.200 × $10^{3}$	2.206 × $10^{3}$	**2.221 × $10^{3}$**	**8.647**
I-CPA [72]	2.224 × $10^{3}$	2.724 × $10^{3}$	6.028 × $10^{3}$	7.261 × $10^{2}$
CMp-DE [67]	2.190 × $10^{3}$	2.222 × $10^{3}$	2.534 × $10^{3}$	2.432 × $10^{2}$
HEST-HEO [68]	2.022 × $10^{3}$	2.219 × $10^{3}$	2.425 × $10^{3}$	2.850 × $10^{2}$
LEA [69]	2.131 × $10^{3}$	2.222 × $10^{3}$	2.503 × $10^{3}$	2.630 × $10^{2}$
PSO [70]	2.104 × $10^{3}$	2.224 × $10^{3}$	2.433 × $10^{3}$	2.326 × $10^{2}$
GWO [57]	3.006 × $10^{3}$	3.216 × $10^{3}$	3.463 × $10^{3}$	3.231 × $10^{2}$
HBO [53]	2.013 × $10^{3}$	2.227 × $10^{3}$	2.512 × $10^{3}$	3.528 × $10^{2}$
LEHBO [58]	2.105 × $10^{3}$	2.220 × $10^{3}$	2.501 × $10^{3}$	2.800 × $10^{2}$
MSIHBO	**2.002 × $10^{3}$**	**2.138 × $10^{3}$**	2.323 × $10^{3}$	2.270 × $10^{2}$

**Table 12:** Experimental results on CEC2022 benchmark function F9.

Algorithm	Best	Mean	Worst	Std
MS-TSA [71]	2.300 × $10^{3}$	2.314 × $10^{3}$	2.659 × $10^{3}$	7.037 × $10^{1}$
I-CPA [72]	2.300 × $10^{3}$	2.684 × $10^{3}$	3.069 × $10^{3}$	1.052 × $10^{2}$
CMp-DE [67]	2.106 × $10^{3}$	2.456 × $10^{3}$	2.698 × $10^{3}$	4.186 × $10^{2}$
HEST-HEO [68]	**2.001 × $10^{3}$**	2.334 × $10^{3}$	2.657 × $10^{3}$	2.284 × $10^{2}$
LEA [69]	2.003 × $10^{3}$	2.300 × $10^{3}$	2.437 × $10^{3}$	9.687 × $10^{1}$
PSO [70]	2.337 × $10^{3}$	2.531 × $10^{3}$	2.768 × $10^{3}$	1.676 × $10^{2}$
GWO [57]	2.213 × $10^{3}$	2.315 × $10^{3}$	2.572 × $10^{3}$	1.817 × $10^{2}$
HBO [53]	2.361 × $10^{3}$	2.659 × $10^{3}$	2.883 × $10^{3}$	1.584 × $10^{2}$
LEHBO [58]	2.296 × $10^{3}$	2.659 × $10^{3}$	2.804 × $10^{3}$	1.025 × $10^{2}$
MSIHBO	2.163 × $10^{3}$	**2.285 × $10^{3}$**	**2.420 × $10^{3}$**	**9.546 × $10^{1}$**

**Table 13:** Experimental results on CEC2022 benchmark function F10.

Algorithm	Best	Mean	Worst	Std
MS-TSA [71]	2.599 × $10^{3}$	2.599 × $10^{3}$	2.603 × $10^{3}$	1.022
I-CPA [72]	2.601 × $10^{3}$	2.805 × $10^{3}$	4.578 × $10^{3}$	4.425 × $10^{2}$
CMp-DE [67]	8.126 × $10^{1}$	8.226 × $10^{1}$	8.531 × $10^{1}$	2.864
HEST-HEO [68]	8.103 × $10^{1}$	8.276 × $10^{1}$	8.488 × $10^{1}$	1.499
LEA [69]	5.605 × $10^{1}$	5.787 × $10^{1}$	6.006 × $10^{1}$	1.549
PSO [70]	1.011 × $10^{3}$	2.051 × $10^{3}$	2.597 × $10^{3}$	3.861 × $10^{2}$
GWO [57]	1.331 × $10^{3}$	2.752 × $10^{3}$	2.977 × $10^{3}$	1.591 × $10^{2}$
HBO [53]	9.316 × $10^{2}$	9.579 × $10^{2}$	9.865 × $10^{2}$	2.022 × $10^{1}$
LEHBO [58]	**3.454 × $10^{1}$**	**3.663 × $10^{1}$**	**4.100 × $10^{1}$**	3.090
MSIHBO	5.537 × $10^{1}$	5.663 × $10^{1}$	5.863 × $10^{1}$	**1.414**

**Table 14:** Experimental results on CEC2022 benchmark function F11.

Algorithm	Best	Mean	Worst	Std
MS-TSA [71]	2.600 × $10^{3}$	**2.600 × $10^{3}$**	**2.600 × $10^{3}$**	**1.349 × $10^{-12}$**
I-CPA [72]	2.600 × $10^{3}$	2.688 × $10^{3}$	3.482 × $10^{3}$	2.339 × $10^{2}$
CMp-DE [67]	2.553 × $10^{3}$	**2.600 × $10^{3}$**	2.723 × $10^{3}$	1.202 × $10^{2}$
HEST-HEO [68]	2.569 × $10^{3}$	2.652 × $10^{3}$	2.696 × $10^{3}$	3.111 × $10^{1}$
LEA [69]	2.564 × $10^{3}$	2.612 × $10^{3}$	2.700 × $10^{3}$	6.223 × $10^{1}$
PSO [70]	3.263 × $10^{3}$	3.348 × $10^{3}$	3.543 × $10^{3}$	1.379 × $10^{2}$
GWO [57]	2.577 × $10^{3}$	2.603 × $10^{3}$	2.692 × $10^{3}$	6.293 × $10^{1}$
HBO [53]	2.513 × $10^{3}$	**2.600 × $10^{3}$**	2.683 × $10^{3}$	5.869 × $10^{1}$
LEHBO [58]	2.534 × $10^{3}$	**2.600 × $10^{3}$**	2.706 × $10^{3}$	7.495 × $10^{1}$
MSIHBO	**2.506 × $10^{3}$**	2.607 × $10^{3}$	2.635 × $10^{3}$	1.980 × $10^{1}$

**Table 15:** Experimental results on CEC2022 benchmark function F12.

Algorithm	Best	Mean	Worst	Std
MS-TSA [71]	2.864 × $10^{3}$	2.866 × $10^{3}$	2.869 × $10^{3}$	**1.263**
I-CPA [72]	2.864 × $10^{3}$	2.878 × $10^{3}$	2.930 × $10^{3}$	1.460 × $10^{1}$
CMp-DE [67]	2.650 × $10^{3}$	2.781 × $10^{3}$	2.861 × $10^{3}$	1.492 × $10^{2}$
HEST-HEO [68]	2.553 × $10^{3}$	2.659 × $10^{3}$	2.736 × $10^{3}$	1.294 × $10^{2}$
LEA [69]	2.526 × $10^{3}$	2.658 × $10^{3}$	2.722 × $10^{3}$	1.386 × $10^{2}$
PSO [70]	2.673 × $10^{3}$	2.750 × $10^{3}$	2.819 × $10^{3}$	1.032 × $10^{2}$
GWO [57]	2.708 × $10^{3}$	2.859 × $10^{3}$	2.955 × $10^{3}$	1.747 × $10^{2}$
HBO [53]	2.679 × $10^{3}$	2.837 × $10^{3}$	2.941 × $10^{3}$	1.853 × $10^{2}$
LEHBO [58]	2.595 × $10^{3}$	2.690 × $10^{3}$	2.753 × $10^{3}$	1.117 × $10^{2}$
MSIHBO	**2.501 × $10^{3}$**	**2.651 × $10^{3}$**	**2.719 × $10^{3}$**	1.541 × $10^{2}$

**Table 16:** Parameter settings of comparative algorithms in the Stage 1 validation experiments.

Algorithm Name	Parameter Settings	
MSIHBO	$max\_self=10,\;a_{max}=2,\;a_{min}=0$	∖
$FGIHBO_{M\;=\;2}$		M=2
$FGIHBO_{M\;=\;3}$		M=3
$FGIHBO_{M\;=\;4}$		M=4
$FGIHBO_{M\;=\;5}$		M=5
$FGIHBO_{M\;=\;6}$		M=6
LEHBO [58]	max_self=10,beta=1.5,step_scaling=0.1	∖
CCIHBO2		M=2
CCIHBO3		M=3
CCIHBO4		M=4
CCIHBO5		M=5
CCIHBO6		M=6

**Table 17:** Experimental results of comparative algorithms on datasets D1–D4.

Dataset	Algorithm	Best	Worst	Mean	Std	Time
D1	MSIHBO	92.24	90.21	91.23	1.02	55.90
	$FGIHBO_{M\;=\;2}$	96.41	92.85	94.63	1.78	44.09
	$FGIHBO_{M\;=\;3}$	97.71	93.56	95.64	2.08	**40.30**
	$FGIHBO_{M\;=\;4}$	98.70	94.07	96.39	2.31	47.14
	$FGIHBO_{M\;=\;5}$	**98.90**	**96.42**	**97.66**	1.24	47.09
	$FGIHBO_{M\;=\;6}$	98.20	94.30	96.25	1.95	52.64
	LEHBO [58]	87.44	84.10	83.77	1.67	62.75
	CCIHBO2	**88.13**	84.57	84.35	1.78	62.19
	CCIHBO3	86.94	**85.04**	83.99	**0.95**	**59.27**
	CCIHBO4	88.03	84.68	**84.36**	1.68	61.22
	CCIHBO5	87.14	84.83	83.99	1.16	67.65
	CCIHBO6	86.94	82.81	82.88	2.07	92.04
D2	MSIHBO	89.34	87.53	88.44	0.91	163.98
	$FGIHBO_{M\;=\;2}$	91.61	87.21	89.41	2.20	159.70
	$FGIHBO_{M\;=\;3}$	94.45	91.45	92.95	1.50	173.63
	$FGIHBO_{M\;=\;4}$	95.60	93.12	94.36	1.24	172.60
	$FGIHBO_{M\;=\;5}$	**97.10**	93.63	95.37	1.74	**154.06**
	$FGIHBO_{M\;=\;6}$	96.62	**94.40**	**95.51**	1.11	184.71
	LEHBO [58]	86.50	83.34	84.92	1.58	195.28
	CCIHBO2	**87.58**	84.29	**85.94**	1.65	**181.05**
	CCIHBO3	85.29	82.47	83.88	1.41	195.95
	CCIHBO4	85.29	83.16	84.23	1.07	224.16
	CCIHBO5	86.01	**84.91**	85.46	**0.55**	185.14
	CCIHBO6	85.71	83.49	84.60	1.11	235.25
D3	MSIHBO	88.36	85.42	86.89	1.47	104.04
	$FGIHBO_{M\;=\;2}$	91.59	87.77	89.68	1.91	**92.64**
	$FGIHBO_{M\;=\;3}$	94.99	91.36	93.18	1.82	96.87
	$FGIHBO_{M\;=\;4}$	95.90	92.12	94.01	1.89	101.88
	$FGIHBO_{M\;=\;5}$	**97.40**	**94.38**	**95.89**	1.51	83.97
	$FGIHBO_{M\;=\;6}$	96.93	93.12	95.03	1.91	100.84
	LEHBO [58]	86.06	82.55	84.31	1.76	149.88
	CCIHBO2	86.28	82.88	84.58	1.70	113.03
	CCIHBO3	86.24	**83.63**	**84.94**	**1.31**	**102.56**
	CCIHBO4	85.99	82.79	84.39	1.60	114.80
	CCIHBO5	**86.31**	83.03	84.67	1.64	116.99
	CCIHBO6	85.18	81.81	83.50	1.69	122.15
D4	MSIHBO	87.92	85.11	86.52	1.41	108.73
	$FGIHBO_{M\;=\;2}$	90.95	88.94	89.95	1.01	99.09
	$FGIHBO_{M\;=\;3}$	94.23	91.88	93.06	1.18	102.37
	$FGIHBO_{M\;=\;4}$	95.78	92.01	93.90	1.89	109.14
	$FGIHBO_{M\;=\;5}$	96.73	**94.72**	95.73	1.01	**89.71**
	$FGIHBO_{M\;=\;6}$	**97.16**	94.50	**95.83**	1.33	107.64
	LEHBO [58]	84.14	81.88	83.01	1.13	151.92
	CCIHBO2	84.55	83.20	83.88	0.68	118.88
	CCIHBO3	**84.78**	**83.99**	**84.39**	**0.40**	123.66
	CCIHBO4	84.21	82.47	83.34	0.87	130.27
	CCIHBO5	84.24	82.96	83.60	0.64	**108.68**
	CCIHBO6	84.04	82.81	83.43	0.62	127.99

**Table 18:** Parameter settings of comparative algorithms in the Stage 2 comprehensive testing experiments.

Algorithm Name	Parameter Settings
MIGWO [1]	w=0.99,F=0.5,pl=0.8,θ=1.5
HWOAG [54]	a=2,b=1
DCSA [55]	$ap_{max}=0.8,\;fl_{min}=0.4,\;fl_{max}=0.4$
SA [56]	T=1000
GWO [57]	*a* decreases linearly from 2 to 0
HBO [53]	max_self=10
LEHBO [58]	max_self=10,beta=1.5,step_scaling=0.1
CCIHBO3	$max\_self=10,\;a_{max}=2,\;a_{min}=0,\;M=3$
MSIHBO	$max\_self=10,\;a_{max}=2,\;a_{min}=0$
$FGIHBO_{M=5}$	$max\_self=10,\;a_{max}=2,\;a_{min}=0,\;M=5$

**Table 19:** Experimental results of comparative algorithms on datasets D5–D11.

Dataset	Algorithm	Best	Worst	Mean	Std
D5	MIGWO [1]	83.18	80.04	81.61	1.11
	HWOAG [54]	81.09	78.68	79.99	**0.85**
	DCSA [55]	81.74	78.05	79.90	1.30
	SA [56]	80.55	77.93	79.24	0.93
	GWO [57]	84.32	81.47	83.40	1.01
	HBO [53]	84.02	81.36	82.69	0.94
	LEHBO [58]	83.97	80.01	82.69	1.40
	CCIHBO3	84.81	81.02	82.92	1.34
	MSIHBO	84.92	81.88	83.40	1.07
	$FGIHBO_{M\;=\;5}$	**96.72**	**94.01**	**95.37**	0.96
D6	MIGWO [1]	83.18	80.98	82.08	1.56
	HWOAG [54]	79.71	75.21	77.46	1.59
	DCSA [55]	81.56	80.66	81.11	**0.32**
	SA [56]	81.06	78.26	79.66	0.99
	GWO [57]	83.77	80.57	82.17	1.13
	HBO [53]	83.00	79.90	81.45	1.10
	LEHBO [58]	83.36	80.16	81.76	1.13
	CCIHBO3	83.22	80.62	81.92	0.92
	MSIHBO	84.76	81.86	83.31	1.03
	$FGIHBO_{M\;=\;5}$	**96.80**	**94.40**	**95.60**	0.85
D7	MIGWO [1]	81.73	79.21	80.47	1.78
	HWOAG [54]	80.49	78.64	79.57	0.65
	DCSA [55]	81.88	79.57	80.73	0.82
	SA [56]	79.74	76.02	77.88	1.32
	GWO [57]	82.40	79.13	80.77	1.16
	HBO [53]	83.11	80.96	82.04	0.76
	LEHBO [58]	83.41	81.64	82.53	**0.63**
	CCIHBO3	83.90	80.11	82.01	1.34
	MSIHBO	85.65	83.02	84.34	0.93
	$FGIHBO_{M\;=\;5}$	**96.96**	**93.48**	**95.22**	1.23
D8	MIGWO [1]	82.61	81.27	81.94	0.95
	HWOAG [54]	80.56	77.73	79.15	1.00
	DCSA [55]	81.29	78.83	80.06	**0.87**
	SA [56]	80.50	77.65	79.08	1.01
	GWO [57]	82.78	80.02	81.40	0.98
	HBO [53]	82.72	80.21	81.47	0.89
	LEHBO [58]	82.92	79.58	81.25	1.18
	CCIHBO3	82.83	79.96	81.40	1.01
	MSIHBO	84.93	82.14	83.54	0.99
	$FGIHBO_{M\;=\;5}$	**96.89**	**93.73**	**95.31**	1.12
D9	MIGWO [1]	82.02	79.63	80.83	1.69
	HWOAG [54]	76.08	72.92	74.50	1.12
	DCSA [55]	81.80	78.41	80.11	1.20
	SA [56]	76.90	73.18	75.04	1.32
	GWO [57]	81.68	77.56	79.62	1.46
	HBO [53]	83.37	79.42	81.40	1.40
	LEHBO [58]	82.69	80.87	81.78	**0.64**
	CCIHBO3	82.66	80.03	81.35	0.93
	MSIHBO	84.44	81.11	82.78	1.18
	$FGIHBO_{M\;=\;5}$	**96.58**	**94.02**	**95.30**	0.91
D10	MIGWO [1]	81.54	79.98	80.76	1.10
	HWOAG [54]	68.96	65.87	67.42	1.09
	DCSA [55]	80.93	77.41	79.17	1.24
	SA [56]	74.68	71.96	73.32	**0.96**
	GWO [57]	80.87	77.74	79.31	1.11
	HBO [53]	81.66	78.42	80.04	1.15
	LEHBO [58]	81.56	76.36	78.96	1.84
	CCIHBO3	81.39	78.05	79.72	1.18
	MSIHBO	83.76	80.21	81.99	1.26
	$FGIHBO_{M\;=\;5}$	**96.49**	**93.27**	**94.88**	1.14
D11	MIGWO [1]	80.34	78.57	79.46	1.25
	HWOAG [54]	73.39	69.38	71.39	1.42
	DCSA [55]	79.85	75.94	77.90	1.38
	SA [56]	75.52	71.21	73.37	1.52
	GWO [57]	79.86	75.22	77.54	1.64
	HBO [53]	80.41	76.86	78.64	1.26
	LEHBO [58]	80.66	77.01	78.84	1.29
	CCIHBO3	79.98	76.42	78.20	1.26
	MSIHBO	82.82	77.16	79.99	2.00
	$FGIHBO_{M\;=\;5}$	**94.20**	**91.34**	**92.77**	**1.01**

**Table 20:** Descriptions of ablation variants.

No.	Variant	Description
1	FGIHBO5	Complete FGIHBO framework
2	MSIHBO	Without feature grouping
3	FGIHBO5 w/o mRMR	Without mRMR pre-filtering
4	FGIHBO5 w/o LHS+EOBL	Without LHS and EOBL initialization
5	FGIHBO5 w/o DynamicPartition	Without dynamic partition strategy
6	FGIHBO5 w/o GWO	Without GWO-guided maintainer update
7	FGIHBO5 w/o SA	Without SA acceptance mechanism

**Table 21:** Classification accuracy (%) of different ablation variants on datasets D1–D4.

Variant	D1 (Colon)	D2 (Lung)	D3 (DLBCL)	D4 (Brain_Tumor_1)
FGIHBO5	**97.25**	**95.34**	**95.86**	**95.11**
MSIHBO	91.46	88.12	86.16	86.43
FGIHBO5 w/o mRMR	75.38	83.90	78.12	80.56
FGIHBO5 w/o LHS+EOBL	88.46	88.78	90.62	86.28
FGIHBO5 w/o DynamicPartition	80.77	86.34	84.38	86.11
FGIHBO5 w/o GWO	76.92	85.46	82.38	80.56
FGIHBO5 w/o SA	80.24	86.34	81.88	77.78

## Data Availability

All datasets employed in this research were obtained from the open-access OpenML database (https://www.openml.org/, accessed on 15 January 2026).
